# Recurrent emergence of SARS-CoV-2 spike deletion H69/V70 and its role in the Alpha variant B.1.1.7

**DOI:** 10.1016/j.celrep.2021.109292

**Published:** 2021-06-08

**Authors:** Bo Meng, Steven A. Kemp, Guido Papa, Rawlings Datir, Isabella A.T.M. Ferreira, Sara Marelli, William T. Harvey, Spyros Lytras, Ahmed Mohamed, Giulia Gallo, Nazia Thakur, Dami A. Collier, Petra Mlcochova, The COVID-19 Genomics UK (COG-UK) Consortium, Samuel C. Robson, Nicholas J. Loman, Thomas R. Connor, Tanya Golubchik, Rocio T. Martinez Nunez, Catherine Ludden, Sally Corden, Ian Johnston, David Bonsall, Colin P. Smith, Ali R. Awan, Giselda Bucca, M. Estee Torok, Kordo Saeed, Jacqui A. Prieto, David K. Jackson, William L. Hamilton, Luke B. Snell, Catherine Moore, Ewan M. Harrison, Sonia Goncalves, Derek J. Fairley, Matthew W. Loose, Joanne Watkins, Rich Livett, Samuel Moses, Roberto Amato, Sam Nicholls, Matthew Bull, Darren L. Smith, Jeff Barrett, David M. Aanensen, Martin D. Curran, Surendra Parmar, Dinesh Aggarwal, James G. Shepherd, Matthew D. Parker, Sharon Glaysher, Matthew Bashton, Anthony P. Underwood, Nicole Pacchiarini, Katie F. Loveson, Kate E. Templeton, Cordelia F. Langford, John Sillitoe, Thushan I. de Silva, Dennis Wang, Dominic Kwiatkowski, Andrew Rambaut, Justin O’Grady, Simon Cottrell, Matthew T.G. Holden, Emma C. Thomson, Husam Osman, Monique Andersson, Anoop J. Chauhan, Mohammed O. Hassan-Ibrahim, Mara Lawniczak, Alex Alderton, Meera Chand, Chrystala Constantinidou, Meera Unnikrishnan, Alistair C. Darby, Julian A. Hiscox, Steve Paterson, Inigo Martincorena, Erik M. Volz, Andrew J. Page, Oliver G. Pybus, Andrew R. Bassett, Cristina V. Ariani, Michael H. Spencer Chapman, Kathy K. Li, Rajiv N. Shah, Natasha G. Jesudason, Yusri Taha, Martin P. McHugh, Rebecca Dewar, Aminu S. Jahun, Claire McMurray, Sarojini Pandey, James P. McKenna, Andrew Nelson, Gregory R. Young, Clare M. McCann, Scott Elliott, Hannah Lowe, Ben Temperton, Sunando Roy, Anna Price, Sara Rey, Matthew Wyles, Stefan Rooke, Sharif Shaaban, Mariateresa de Cesare, Laura Letchford, Siona Silveira, Emanuela Pelosi, Eleri Wilson-Davies, Myra Hosmillo, Áine O’Toole, Andrew R. Hesketh, Richard Stark, Louis du Plessis, Chris Ruis, Helen Adams, Yann Bourgeois, Stephen L. Michell, Dimitris Gramatopoulos, Jonathan Edgeworth, Judith Breuer, John A. Todd, Christophe Fraser, David Buck, Michaela John, Gemma L. Kay, Steve Palmer, Sharon J. Peacock, David Heyburn, Danni Weldon, Esther Robinson, Alan McNally, Peter Muir, Ian B. Vipond, John Boyes, Venkat Sivaprakasam, Tranprit Salluja, Samir Dervisevic, Emma J. Meader, Naomi R. Park, Karen Oliver, Aaron R. Jeffries, Sascha Ott, Ana da Silva Filipe, David A. Simpson, Chris Williams, Jane A.H. Masoli, Bridget A. Knight, Christopher R. Jones, Cherian Koshy, Amy Ash, Anna Casey, Andrew Bosworth, Liz Ratcliffe, Li Xu-McCrae, Hannah M. Pymont, Stephanie Hutchings, Lisa Berry, Katie Jones, Fenella Halstead, Thomas Davis, Christopher Holmes, Miren Iturriza-Gomara, Anita O. Lucaci, Paul Anthony Randell, Alison Cox, Pinglawathee Madona, Kathryn Ann Harris, Julianne Rose Brown, Tabitha W. Mahungu, Dianne Irish-Tavares, Tanzina Haque, Jennifer Hart, Eric Witele, Melisa Louise Fenton, Steven Liggett, Clive Graham, Emma Swindells, Jennifer Collins, Gary Eltringham, Sharon Campbell, Patrick C. McClure, Gemma Clark, Tim J. Sloan, Carl Jones, Jessica Lynch, Ben Warne, Steven Leonard, Jillian Durham, Thomas Williams, Sam T. Haldenby, Nathaniel Storey, Nabil-Fareed Alikhan, Nadine Holmes, Christopher Moore, Matthew Carlile, Malorie Perry, Noel Craine, Ronan A. Lyons, Angela H. Beckett, Salman Goudarzi, Christopher Fearn, Kate Cook, Hannah Dent, Hannah Paul, Robert Davies, Beth Blane, Sophia T. Girgis, Mathew A. Beale, Katherine L. Bellis, Matthew J. Dorman, Eleanor Drury, Leanne Kane, Sally Kay, Samantha McGuigan, Rachel Nelson, Liam Prestwood, Shavanthi Rajatileka, Rahul Batra, Rachel J. Williams, Mark Kristiansen, Angie Green, Anita Justice, Adhyana I.K. Mahanama, Buddhini Samaraweera, Nazreen F. Hadjirin, Joshua Quick, Radoslaw Poplawski, Leanne M. Kermack, Nicola Reynolds, Grant Hall, Yasmin Chaudhry, Malte L. Pinckert, Iliana Georgana, Robin J. Moll, Alicia Thornton, Richard Myers, Joanne Stockton, Charlotte A. Williams, Wen C. Yew, Alexander J. Trotter, Amy Trebes, George MacIntyre-Cockett, Alec Birchley, Alexander Adams, Amy Plimmer, Bree Gatica-Wilcox, Caoimhe McKerr, Ember Hilvers, Hannah Jones, Hibo Asad, Jason Coombes, Johnathan M. Evans, Laia Fina, Lauren Gilbert, Lee Graham, Michelle Cronin, Sara Kumziene-Summerhayes, Sarah Taylor, Sophie Jones, Danielle C. Groves, Peijun Zhang, Marta Gallis, Stavroula F. Louka, Igor Starinskij, Chris Jackson, Marina Gourtovaia, Gerry Tonkin-Hill, Kevin Lewis, Jaime M. Tovar-Corona, Keith James, Laura Baxter, Mohammad T. Alam, Richard J. Orton, Joseph Hughes, Sreenu Vattipally, Manon Ragonnet-Cronin, Fabricia F. Nascimento, David Jorgensen, Olivia Boyd, Lily Geidelberg, Alex E. Zarebski, Jayna Raghwani, Moritz U.G. Kraemer, Joel Southgate, Benjamin B. Lindsey, Timothy M. Freeman, Jon-Paul Keatley, Joshua B. Singer, Leonardo de Oliveira Martins, Corin A. Yeats, Khalil Abudahab, Ben E.W. Taylor, Mirko Menegazzo, John Danesh, Wendy Hogsden, Sahar Eldirdiri, Anita Kenyon, Jenifer Mason, Trevor I. Robinson, Alison Holmes, James Price, John A. Hartley, Tanya Curran, Alison E. Mather, Giri Shankar, Rachel Jones, Robin Howe, Sian Morgan, Elizabeth Wastenge, Michael R. Chapman, Siddharth Mookerjee, Rachael Stanley, Wendy Smith, Timothy Peto, David Eyre, Derrick Crook, Gabrielle Vernet, Christine Kitchen, Huw Gulliver, Ian Merrick, Martyn Guest, Robert Munn, Declan T. Bradley, Tim Wyatt, Charlotte Beaver, Luke Foulser, Sophie Palmer, Carol M. Churcher, Ellena Brooks, Kim S. Smith, Katerina Galai, Georgina M. McManus, Frances Bolt, Francesc Coll, Lizzie Meadows, Stephen W. Attwood, Alisha Davies, Elen De Lacy, Fatima Downing, Sue Edwards, Garry P. Scarlett, Sarah Jeremiah, Nikki Smith, Danielle Leek, Sushmita Sridhar, Sally Forrest, Claire Cormie, Harmeet K. Gill, Joana Dias, Ellen E. Higginson, Mailis Maes, Jamie Young, Michelle Wantoch, Dorota Jamrozy, Stephanie Lo, Minal Patel, Verity Hill, Claire M. Bewshea, Sian Ellard, Cressida Auckland, Ian Harrison, Chloe Bishop, Vicki Chalker, Alex Richter, Andrew Beggs, Angus Best, Benita Percival, Jeremy Mirza, Oliver Megram, Megan Mayhew, Liam Crawford, Fiona Ashcroft, Emma Moles-Garcia, Nicola Cumley, Richard Hopes, Patawee Asamaphan, Marc O. Niebel, Rory N. Gunson, Amanda Bradley, Alasdair Maclean, Guy Mollett, Rachel Blacow, Paul Bird, Thomas Helmer, Karlie Fallon, Julian Tang, Antony D. Hale, Louissa R. Macfarlane-Smith, Katherine L. Harper, Holli Carden, Nicholas W. Machin, Kathryn A. Jackson, Shazaad S.Y. Ahmad, Ryan P. George, Lance Turtle, Elaine O’Toole, Joanne Watts, Cassie Breen, Angela Cowell, Adela Alcolea-Medina, Themoula Charalampous, Amita Patel, Lisa J. Levett, Judith Heaney, Aileen Rowan, Graham P. Taylor, Divya Shah, Laura Atkinson, Jack C.D. Lee, Adam P. Westhorpe, Riaz Jannoo, Helen L. Lowe, Angeliki Karamani, Leah Ensell, Wendy Chatterton, Monika Pusok, Ashok Dadrah, Amanda Symmonds, Graciela Sluga, Zoltan Molnar, Paul Baker, Stephen Bonner, Sarah Essex, Edward Barton, Debra Padgett, Garren Scott, Jane Greenaway, Brendan A.I. Payne, Shirelle Burton-Fanning, Sheila Waugh, Veena Raviprakash, Nicola Sheriff, Victoria Blakey, Lesley-Anne Williams, Jonathan Moore, Susanne Stonehouse, Louise Smith, Rose K. Davidson, Luke Bedford, Lindsay Coupland, Victoria Wright, Joseph G. Chappell, Theocharis Tsoleridis, Jonathan Ball, Manjinder Khakh, Vicki M. Fleming, Michelle M. Lister, Hannah C. Howson-Wells, Louise Berry, Tim Boswell, Amelia Joseph, Iona Willingham, Nichola Duckworth, Sarah Walsh, Emma Wise, Nathan Moore, Matilde Mori, Nick Cortes, Stephen Kidd, Rebecca Williams, Laura Gifford, Kelly Bicknell, Sarah Wyllie, Allyson Lloyd, Robert Impey, Cassandra S. Malone, Benjamin J. Cogger, Nick Levene, Lynn Monaghan, Alexander J. Keeley, David G. Partridge, Mohammad Raza, Cariad Evans, Kate Johnson, Emma Betteridge, Ben W. Farr, Scott Goodwin, Michael A. Quail, Carol Scott, Lesley Shirley, Scott A.J. Thurston, Diana Rajan, Iraad F. Bronner, Louise Aigrain, Nicholas M. Redshaw, Stefanie V. Lensing, Shane McCarthy, Alex Makunin, Carlos E. Balcazar, Michael D. Gallagher, Kathleen A. Williamson, Thomas D. Stanton, Michelle L. Michelsen, Joanna Warwick-Dugdale, Robin Manley, Audrey Farbos, James W. Harrison, Christine M. Sambles, David J. Studholme, Angie Lackenby, Tamyo Mbisa, Steven Platt, Shahjahan Miah, David Bibby, Carmen Manso, Jonathan Hubb, Gavin Dabrera, Mary Ramsay, Daniel Bradshaw, Ulf Schaefer, Natalie Groves, Eileen Gallagher, David Lee, David Williams, Nicholas Ellaby, Hassan Hartman, Nikos Manesis, Vineet Patel, Juan Ledesma, Katherine A. Twohig, Elias Allara, Clare Pearson, Jeffrey K.J. Cheng, Hannah E. Bridgewater, Lucy R. Frost, Grace Taylor-Joyce, Paul E. Brown, Lily Tong, Alice Broos, Daniel Mair, Jenna Nichols, Stephen N. Carmichael, Katherine L. Smollett, Kyriaki Nomikou, Elihu Aranday-Cortes, Natasha Johnson, Seema Nickbakhsh, Edith E. Vamos, Margaret Hughes, Lucille Rainbow, Richard Eccles, Charlotte Nelson, Mark Whitehead, Richard Gregory, Matthew Gemmell, Claudia Wierzbicki, Hermione J. Webster, Chloe L. Fisher, Adrian W. Signell, Gilberto Betancor, Harry D. Wilson, Gaia Nebbia, Flavia Flaviani, Alberto C. Cerda, Tammy V. Merrill, Rebekah E. Wilson, Marius Cotic, Nadua Bayzid, Thomas Thompson, Erwan Acheson, Steven Rushton, Sarah O’Brien, David J. Baker, Steven Rudder, Alp Aydin, Fei Sang, Johnny Debebe, Sarah Francois, Tetyana I. Vasylyeva, Marina Escalera Zamudio, Bernardo Gutierrez, Angela Marchbank, Joshua Maksimovic, Karla Spellman, Kathryn McCluggage, Mari Morgan, Robert Beer, Safiah Afifi, Trudy Workman, William Fuller, Catherine Bresner, Adrienn Angyal, Luke R. Green, Paul J. Parsons, Rachel M. Tucker, Rebecca Brown, Max Whiteley, James Bonfield, Christoph Puethe, Andrew Whitwham, Jennifier Liddle, Will Rowe, Igor Siveroni, Thanh Le-Viet, Amy Gaskin, Rob Johnson, Irina Abnizova, Mozam Ali, Laura Allen, Ralph Anderson, Cristina Ariani, Siobhan Austin-Guest, Sendu Bala, Jeffrey Barrett, Andrew Bassett, Kristina Battleday, James Beal, Mathew Beale, Sam Bellany, Tristram Bellerby, Katie Bellis, Duncan Berger, Matt Berriman, Paul Bevan, Simon Binley, Jason Bishop, Kirsty Blackburn, Nick Boughton, Sam Bowker, Timothy Brendler-Spaeth, Iraad Bronner, Tanya Brooklyn, Sarah Kay Buddenborg, Robert Bush, Catarina Caetano, Alex Cagan, Nicola Carter, Joanna Cartwright, Tiago Carvalho Monteiro, Liz Chapman, Tracey-Jane Chillingworth, Peter Clapham, Richard Clark, Adrian Clarke, Catriona Clarke, Daryl Cole, Elizabeth Cook, Maria Coppola, Linda Cornell, Clare Cornwell, Craig Corton, Abby Crackett, Alison Cranage, Harriet Craven, Sarah Craw, Mark Crawford, Tim Cutts, Monika Dabrowska, Matt Davies, Joseph Dawson, Callum Day, Aiden Densem, Thomas Dibling, Cat Dockree, David Dodd, Sunil Dogga, Matthew Dorman, Gordon Dougan, Martin Dougherty, Alexander Dove, Lucy Drummond, Monika Dudek, Laura Durrant, Elizabeth Easthope, Sabine Eckert, Pete Ellis, Ben Farr, Michael Fenton, Marcella Ferrero, Neil Flack, Howerd Fordham, Grace Forsythe, Matt Francis, Audrey Fraser, Adam Freeman, Anastasia Galvin, Maria Garcia-Casado, Alex Gedny, Sophia Girgis, James Glover, Oliver Gould, Andy Gray, Emma Gray, Coline Griffiths, Yong Gu, Florence Guerin, Will Hamilton, Hannah Hanks, Ewan Harrison, Alexandria Harrott, Edward Harry, Julia Harvison, Paul Heath, Anastasia Hernandez-Koutoucheva, Rhiannon Hobbs, Dave Holland, Sarah Holmes, Gary Hornett, Nicholas Hough, Liz Huckle, Lena Hughes-Hallet, Adam Hunter, Stephen Inglis, Sameena Iqbal, Adam Jackson, David Jackson, Carlos Jimenez Verdejo, Matthew Jones, Kalyan Kallepally, Keely Kay, Jon Keatley, Alan Keith, Alison King, Lucy Kitchin, Matt Kleanthous, Martina Klimekova, Petra Korlevic, Ksenia Krasheninnkova, Greg Lane, Cordelia Langford, Adam Laverack, Katharine Law, Stefanie Lensing, Amanah Lewis-Wade, Jennifer Liddle, Quan Lin, Sarah Lindsay, Sally Linsdell, Rhona Long, Jamie Lovell, Jon Lovell, James Mack, Mark Maddison, Aleksei Makunin, Irfan Mamun, Jenny Mansfield, Neil Marriott, Matt Martin, Matthew Mayho, Jo McClintock, Sandra McHugh, Liz MapcMinn, Carl Meadows, Emily Mobley, Robin Moll, Maria Morra, Leanne Morrow, Kathryn Murie, Sian Nash, Claire Nathwani, Plamena Naydenova, Alexandra Neaverson, Ed Nerou, Jon Nicholson, Tabea Nimz, Guillaume G. Noell, Sarah O’Meara, Valeriu Ohan, Charles Olney, Doug Ormond, Agnes Oszlanczi, Yoke Fei Pang, Barbora Pardubska, Naomi Park, Aaron Parmar, Gaurang Patel, Maggie Payne, Sharon Peacock, Arabella Petersen, Deborah Plowman, Tom Preston, Michael Quail, Richard Rance, Suzannah Rawlings, Nicholas Redshaw, Joe Reynolds, Mark Reynolds, Simon Rice, Matt Richardson, Connor Roberts, Katrina Robinson, Melanie Robinson, David Robinson, Hazel Rogers, Eduardo Martin Rojo, Daljit Roopra, Mark Rose, Luke Rudd, Ramin Sadri, Nicholas Salmon, David Saul, Frank Schwach, Phil Seekings, Alison Simms, Matt Sinnott, Shanthi Sivadasan, Bart Siwek, Dale Sizer, Kenneth Skeldon, Jason Skelton, Joanna Slater-Tunstill, Lisa Sloper, Nathalie Smerdon, Chris Smith, Christen Smith, James Smith, Katie Smith, Michelle Smith, Sean Smith, Tina Smith, Leighton Sneade, Carmen Diaz Soria, Catarina Sousa, Emily Souster, Andrew Sparkes, Michael Spencer-Chapman, Janet Squares, Robert Stanley, Claire Steed, Tim Stickland, Ian Still, Mike Stratton, Michelle Strickland, Allen Swann, Agnieszka Swiatkowska, Neil Sycamore, Emma Swift, Edward Symons, Suzanne Szluha, Emma Taluy, Nunu Tao, Katy Taylor, Sam Taylor, Stacey Thompson, Mark Thompson, Mark Thomson, Nicholas Thomson, Scott Thurston, Dee Toombs, Benjamin Topping, Jaime Tovar-Corona, Daniel Ungureanu, James Uphill, Jana Urbanova, Philip Jansen Van, Valerie Vancollie, Paul Voak, Danielle Walker, Matthew Walker, Matt Waller, Gary Ward, Charlie Weatherhogg, Niki Webb, Alan Wells, Eloise Wells, Luke Westwood, Theo Whipp, Thomas Whiteley, Georgia Whitton, Sara Widaa, Mia Williams, Mark Wilson, Sean Wright, Lidia M. Duncan, Alessandro M. Carabelli, Julia C. Kenyon, Andrew M. Lever, Anna De Marco, Christian Saliba, Katja Culap, Elisabetta Cameroni, Nicholas J. Matheson, Luca Piccoli, Davide Corti, Leo C. James, David L. Robertson, Dalan Bailey, Ravindra K. Gupta

**Affiliations:** 1 Cambridge Institute of Therapeutic Immunology & Infectious Disease (CITIID), Cambridge, UK; 2 Department of Medicine, University of Cambridge, Cambridge, UK; 3 Division of Infection and Immunity, University College London, London, UK; 4 MRC – Laboratory of Molecular Biology, Cambridge, UK; 5 Institute of Biodiversity, Animal Health and Comparative Medicine, University of Glasgow, Glasgow, UK; 6 MRC – University of Glasgow Centre for Virus Research, Glasgow, UK; 7 Pirbright Institute, Woking, Surrey, UK; 8 https://www.cogconsortium.uk; 9 Department of Microbiology and Immunology, Yong Loo Lin School of Medicine, National University of Singapore, Singapore, Singapore; 10 Department of Medicine, Yong Loo Lin School of Medicine, National University of Singapore, Singapore, Singapore; 11 Humabs Biomed SA, a subsidiary of Vir Biotechnology, 6500 Bellinzona, Switzerland; 12 NHS Blood and Transplant, Cambridge, UK; 13 Africa Health Research Institute, Durban, South Africa

**Keywords:** SARS-CoV-2, COVID-19, antibody escape, neutralizing antibodies, infectivity, spike mutation, Alpha variant, resistance, B.1.1.7, deletion

## Abstract

We report severe acute respiratory syndrome coronavirus 2 (SARS-CoV-2) spike ΔH69/V70 in multiple independent lineages, often occurring after acquisition of receptor binding motif replacements such as N439K and Y453F, known to increase binding affinity to the ACE2 receptor and confer antibody escape. *In vitro*, we show that, although ΔH69/V70 itself is not an antibody evasion mechanism, it increases infectivity associated with enhanced incorporation of cleaved spike into virions. ΔH69/V70 is able to partially rescue infectivity of spike proteins that have acquired N439K and Y453F escape mutations by increased spike incorporation. In addition, replacement of the H69 and V70 residues in the Alpha variant B.1.1.7 spike (where ΔH69/V70 occurs naturally) impairs spike incorporation and entry efficiency of the B.1.1.7 spike pseudotyped virus. Alpha variant B.1.1.7 spike mediates faster kinetics of cell-cell fusion than wild-type Wuhan-1 D614G, dependent on ΔH69/V70. Therefore, as ΔH69/V70 compensates for immune escape mutations that impair infectivity, continued surveillance for deletions with functional effects is warranted.

## Highlights

• Spike ΔH69/V70 does not confer escape from antibodies
• Spike ΔH69/V70 increases cleaved S2 and spike infectivity
• B.1.1.7 requires ΔH69/V70 for efficient cleaved spike incorporation and infectivity
• B.1.1.7 spike requires ΔH69/V70 for rapid syncytium formation

## Graphical abstract

**Figure:**
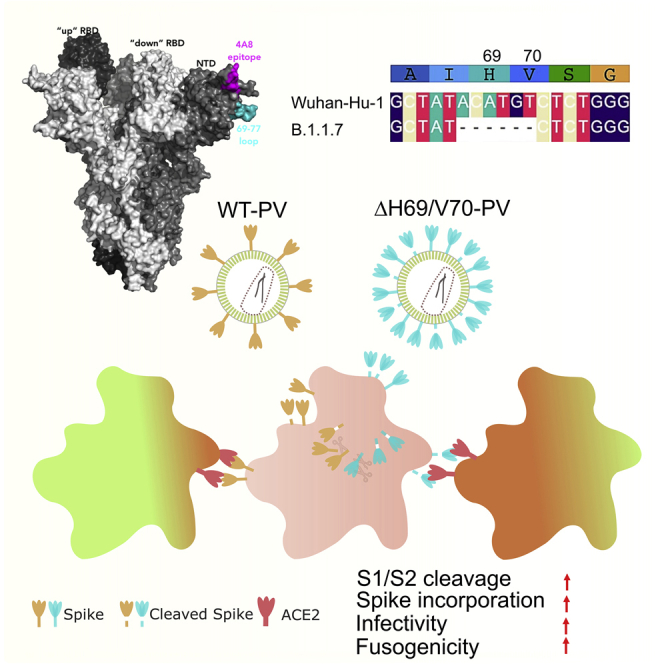

## Teaser

Meng et al. report that the SARS-CoV-2 spike ΔH69/V70 has arisen multiple times. The deletion increases entry efficiency associated with increased cleaved spike in virions and can compensate for loss of infectivity. The B.1.1.7 spike requires ΔH69/V70 for efficient cell entry and cell-cell fusion activity.

## Introduction

Severe acute respiratory syndrome coronavirus 2 (SARS-CoV-2) spike surface glycoprotein engagement of human angiotensin-converting enzyme (hACE2) is essential for virus entry and infection (Zhou et al., 2020), and the receptor is found in the respiratory and gastrointestinal tracts (Sungnak et al., 2020). Despite this critical interaction and the imposed constraints, it appears that the receptor binding domain (RBD) is relatively tolerant to mutations (Starr et al., 2020b; Thomson et al., 2020), raising the real possibility of virus escape from past infection or vaccine-induced immunity (Cele et al., 2021; Collier et al., 2021; Gupta, 2021; Madhi et al., 2021) and monoclonal antibody treatments (Starr et al., 2021). Spike mutants exhibiting reduced susceptibility to neutralizing antibodies have been identified in *in vitro* screens (Greaney et al., 2021a, 2021b; Starr et al., 2020a), and some of these mutations have been found in clinical isolates (Choi et al., 2020).

Studying chronic SARS-CoV-2 infection can give insights into virus evolution that would require many chains of acute transmission to generate. This is because the majority of infections arise as a result of early transmission during pre- or asymptomatic phases prior to peak adaptive responses, and virus adaptation is not observed because the virus is usually cleared by the immune response (He et al., 2020; Mlcochova et al., 2020). We recently documented *de novo* emergence of antibody evasion mutations mediated by spike gene mutations in an individual treated with convalescent plasma (CP) (Kemp et al., 2021). In addition, a chronically infected immune-suppressed individual has been reported recently in Russia with emergence of Y453F along with ΔH69/V70 (Bazykin et al., 2021). Deletions in other parts of the N-terminal domain (NTD) have been reported to arise in chronic infection (Choi et al., 2020) and reduce sensitivity to NTD-specific neutralizing antibodies (McCallum et al., 2021; McCarthy et al., 2021).

Here we analyze global SARS-CoV-2 data and find that ΔH69/V70 occurs independently, often emerging after a significant RBD amino acid replacement, such as Y453F and N439K, which are known to facilitate neutralizing antibody escape or alter ACE2 binding while incurring an infectivity defect, according to some reports (Motozono et al., 2021; Thomson et al., 2021). Although structural modeling indicates that H69/V70 is in an exposed loop that contracts after deletion, potentially altering an antigenic site, we report that the ΔH69/V70 does not confer significantly reduced susceptibility to convalescent sera or monoclonal antibodies (mAbs). Functionally, we find that ΔH69/V70 does increase spike infectivity and compensates for an infectivity defect resulting from the RBD replacements N439K and Y453F. The infectivity increase is driven by higher levels of spike incorporation into virions. We demonstrate that the deletion is required for optimal infectivity of the 501Y.V1 (B.1.1.7) spike protein. We show that, although B.1.1.7 and the wild-type (WT) spike pseudotyped virus (PV) have similar infectivity on a range of target cell types, the B.1.1.7 spike protein alone induces more rapid cell-cell fusion and formation of multinucleated cells. Repair of the two amino acids not only leads to reduced B.1.1.7 spike incorporation into virions and impaired infectivity but also reduces cell-cell fusion kinetics back to WT levels.

## Results

### Multiple occurrences and transmission of spike ΔH69/V70 with and without spike mutations

**Figure 1:**
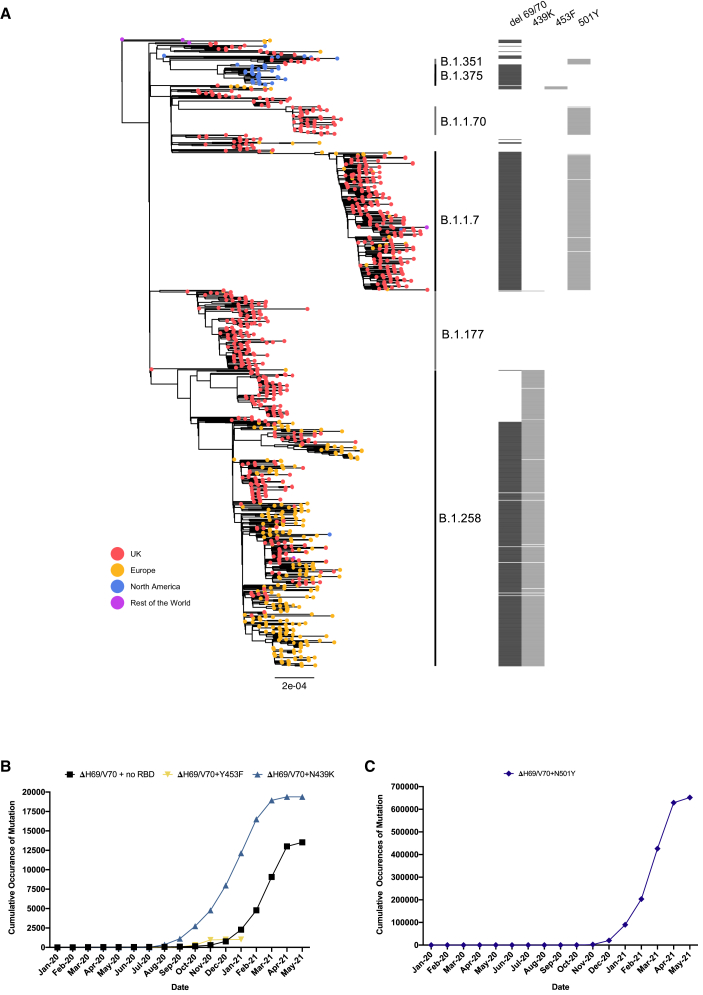
Global snapshot of SARS-CoV-2 lineages associated with ΔH69/V70 and RBM mutations (A) Maximum-likelihood phylogeny of global SARS-CoV-2 whole-genome sequences, highlighting those with specific mutations in spike: ΔH69/V70, N439K, Y453F, and N501Y. The tree is subsampled, and tips are colored by geographic region (see key). Grey bars on the right show the presence or absence of the ΔH69/V70 and amino acid variants N439K, Y453F, and N501Y. Pangolin lineages are shown. (B and C) Cumulative occurrences of SARS-CoV-2 sequences with ΔH69/V70 by month for (B) ΔH69/V70 with or without N439K/Y453F and (C) ΔH69/V70 with N501Y. Indicated frequencies are by month, and all data were collected from the GISAID database (http://gisaid.org, accessed May 21, 2021) and sorted by reporting country and sampling date.

The deletion H69/V70 is present in over 600,000 SARS-CoV-2 genome sequences worldwide and has seen global expansion, particularly across much of Europe, Africa, and Asia (Figure 1). ΔH69/V70 is observed in multiple global lineages (Figure 1A). Although variants with deletions in this region of spike are observed in GISAID (Shu and McCauley, 2017), the earliest unambiguous sequences that included the ΔH69/V70 were detected on a D614 background in January 2020 (United States and Thailand). The earliest ΔH69/V70 detected on a D614G background occurred in Sweden in April 2020. Prevalence of ΔH69/V70 has increased since August 2020 (Figure 1B). Further analysis of sequences revealed, first, that single deletion of H69 or V70 was uncommon and, second, that some lineages of ΔH69/V70 alone were present as well as ΔH69/V70 in the context of other mutations in spike, specifically those in the receptor binding motif (RBM; Figure 1).

### ΔH69/V70 is not a neutralizing antibody escape mechanism

**Figure 2:**
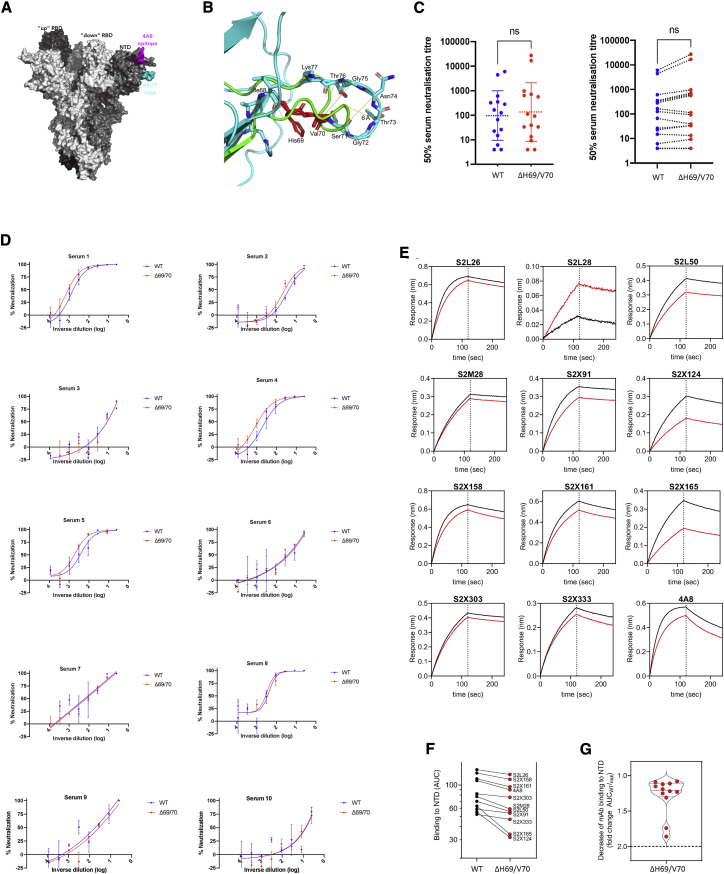
Spike ΔH69/V70 does not reduce sensitivity to neutralizing antibodies (A) Surface representation of the spike homotrimer in the open conformation (PDB: 7C2L), with each monomer shown in different shades of gray. On the monomer shown positioned to the right, the exposed loop consisting of residues 69–77 is shown in cyan and the neutralizing antibody (4A8)-binding NTD epitope in magenta. (B) Prediction of conformational change in the spike NTD because of deletion of residues His69 and Val70. The pre-deletion structure is shown in cyan, except for residues 69 and 70, which are shown in red. The predicted post-deletion structure is shown in green. Residues 66–77 of the pre-deletion structure are shown in stick representation and colored by atom (nitrogen in blue, oxygen in coral). Yellow lines connect aligned residues 66–77 of the pre- and post-deletion structures, and the distance of 6 Å between aligned alpha carbons of Thr73 in the pre- and post-deletion conformation is labeled. (C) Neutralization of spike ΔH69/V70 PV and WT (D614G background) by convalescent sera from 15 donors. GMT (geometric mean titer) with SD presented is representative of two independent experiments, each with two technical repeats. Wilcoxon matched-pairs signed-rank test; ns, not significant. (D) Ten example neutralization curves. Indicated is serum log_10_ inverse dilution against percent neutralization. Data points represent means of technical replicates, and error bars represent SD. Curves are representative of two independent experiments. (E–G) Kinetics of binding to WT and ΔH69/V70 NTD of 12 NTD-specific mAbs. (E) Biolayer interferometry analysis of binding to wild-type (WT; black) and WT ΔH69/V70 (red) NTDs by 12 NTD-targeting mAbs. Dotted lines separate the association phase from the dissociation phase. Shown is 1 of 2 independent experiments. (F) Side-by-side comparison of binding to WT (black) and ΔH69/V70 (red) NTDs by 11 NTD-targeting mAbs. Binding is shown as area under the curve (AUC). The S2L28 mAb is not shown because of too little response measured (<0.10 nm). (G) Binding to NTD of the 11 mAbs shown in (B), expressed as fold change of the AUC of the WT compared with ΔH69/V70. Data are representative of two independent experiments.

We hypothesized that ΔH69/V70 might confer reduced susceptibility to neutralizing antibodies. We first examined the protein structural context of ΔH69/V70 for clues regarding alterations in epitopes (Figures 2A and 2B). In the absence of experimentally derived structural data for ΔH69/V70, the protein structure of the NTD possessing the double deletion was modeled *in silico*. The ΔH69/V70 was predicted to alter the conformation of a protruding loop comprising residues 69–76, pulling it in toward the NTD (Figure 2B). In the post-deletion structural model, the positions of the alpha carbons of residues on either side of the deleted residues, Ile68 and Ser71, were each predicted to occupy positions 2.9 Å from the positions of His69 and Val70 in the pre-deletion structure. Concurrently, the positions of Ser71, Gly72, Thr73, Asn74, and Gly75 are predicted to have changed by 6.5 Å, 6.7 Å, 6.0 Å, 6.2 Å, and 8 Å, respectively, with the overall effect of these residues moving inward, resulting in a less dramatically protruding loop.

This predicted change in the surface of spike could be consistent with antibody evasion. To test this, we explored whether ΔH69/V70 conferred reduced susceptibility to neutralizing antibodies in sera from 15 recovered individuals (Figures 2C and 2D). We performed serial dilutions of sera before mixing with lentiviral particles pseudotyped with spike proteins with and without ΔH69/V70 (with virus input normalized for infectivity). We plotted infection of target cells as a function of serum dilution (Figure 2D). All but two sera demonstrated clear titratable neutralization of WT and ΔH69/V70 virus. There was no overall change in susceptibility to serum neutralization for ΔH69/V70 relative to the WT (Figure 2C), but there was a proportion of individuals with slightly increased neutralization sensitivity of ΔH69/V70 (Figures 2C and 2D). To further explore the role of ΔH69/V70 in inducing immune escape, we tested the binding of 12 NTD mAbs to the WT and ΔH69/V70 NTD by biolayer interferometry (Figures 2E–2G). All NTD mAbs showed less than a 2-fold decrease in binding to ΔH69/V70 compared with the WT. These data suggest that ΔH69/V70 does not represent an important antibody escape mechanism.

### ΔH69/V70 spike enhances infectivity associated with increased cleaved S incorporation

**Figure 3:**
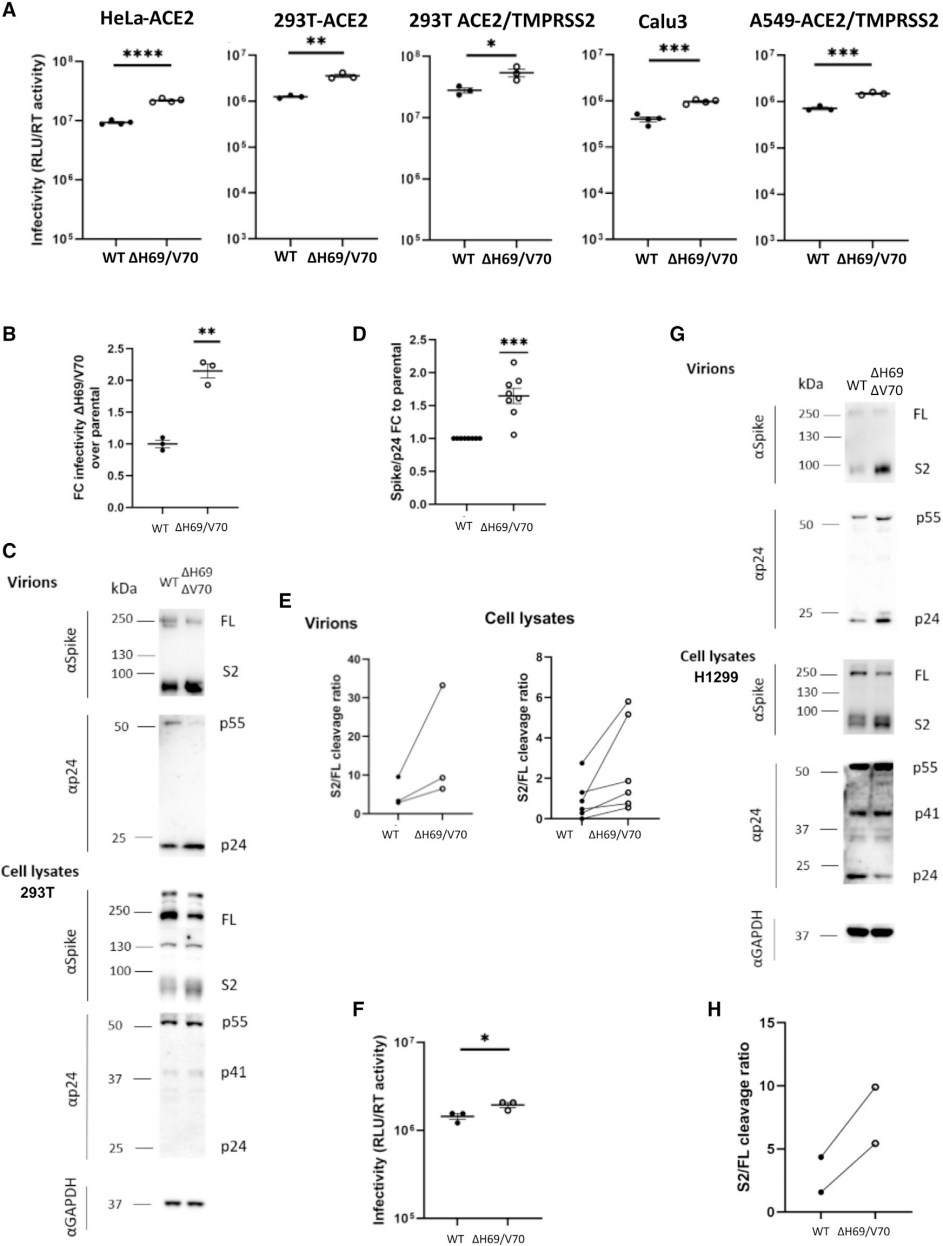
Spike ΔH69/V70 enhances entry and is accompanied by increased spike S2 incorporation into virions (A) Single-round infectivity on different cell targets by spike ΔH69/V70 versus the WT PV produced in HEK293T cells. Data are representative of at least three independent experiments. Data are shown with mean and SEM, and the statistics were performed using unpaired Student’s t test. (B) Infectivity of ΔH69/V70 PV on target HeLa cells transduced with ACE2, expressed as fold change relative to the WT. Mean and SEM are shown; one-sample t test, ^∗∗^p < 0.01 (C–E) Western blots and quantification of virions with infectivity shown in (B) and of cell lysates of HEK293T producer cells following transfection with plasmids expressing lentiviral vectors and SARS-CoV-2 S ΔH69/V70 versus the WT (all with D614G), probed with antibodies for HIV-1 p24 and SARS-Cov-2 S2 (C). (D) Quantification of spike:p24 ratio in supernatants for WT virus with ΔH69/V70 versus the WT alone across multiple replicate experiments. Mean and SEM are shown; one-sample t test, ^∗∗∗^p < 0.001. (E). Quantification of cleaved S2 spike: FL spike for WT virus with ΔH69/V70 versus the WT alone in virions and cell lysates. Each data point represents a single experiment. (F) Infectivity of ΔH69/V70 PV produced in H1299 lung epithelial cells on target HEK293T cells transiently expressing ACE2 and TMPRSS2. The statistical analysis was performed using unpaired Student’s t test. (G) Western blots of virions and cell lysates of H1299 lung epithelial producer cells following transfection with plasmids expressing lentiviral vectors and SARS-CoV-2 S ΔH69/V70 versus the WT (all with D614G). (H) Quantification of the S2:FL ratio in purified virions from H1299 lung epithelial producer cells. Data from at least two independent experiments are shown. RLU: relative light units; RT: reverse transcriptase.

We hypothesized that the deletion might, alternatively, enhance virus infectivity. In the absence of virus isolates, we used a lentiviral PV approach to test the effect of ΔH69/V70 on virus spike protein-mediated entry. D614G bearing Wuhan-1 spike-expressing DNA plasmid (WT) was co-transfected in HEK293T producer cells along with plasmids encoding a lentiviral capsid and genome for luciferase. Infectivity was adjusted for input reverse-transcriptase activity; we observed a 2-fold increase in PV infectivity of ΔH69/V70 compared with the WT in HeLa cervical epithelial cells stably expressing human ACE2 (Figures 3A and 3B). We observed similar fold increases with ΔH69/V70 in a range of other target cells in the context of overexpression of ACE2/TMPRSS2 (HEK293T cells transiently transfected with ACE2, or ACE2 and TMPRSS2 and A549 lung cells stably expressing ACE2 and TMPRSS2; Rihn et al., 2021) or endogenous levels of receptors in Calu-3 lung adenocarcinoma cells (Figure 3A).

Western blotting for S2 spike indicated a higher amount of cleaved spike in ΔH69/V70 bearing virions and in the HEK293T producer cell lysates. We also noted a corresponding reduction in uncleaved full-length (FL) spike (Figure 3C). Densitometric analysis of spike and p24 from western blots in multiple experiments showed almost a 2-fold increase in the spike:p24 ratio as well as an increased ratio in S2:FL cleavage for ΔH69/V70, indicating that increased spike incorporation into virions might explain the increase in infectivity (Figures 3D and 3E). To verify that this increase in S from producer cells was not specific to HEK293T cells, we also transfected the human lung epithelial cell line H1299 (Zhang et al., 2020) with spike and lentiviral packaging plasmids. We again observed that viruses from these cells had 2-fold increased infectivity in target cells (Figure 3F). In addition, the increased total and cleaved S levels were recapitulated in the cell lysates and purified virions from these lung cells (Figures 3G and 3H). Therefore, we conclude that the increased S cleavage and its incorporation observed in producer cells and pseudotyped virions is a generalized phenomenon for ΔH69/V70 S. To explore whether D614G was required for this enhanced spike cleavage and infectivity, we generated PVs bearing D614 spike with and without ΔH69/V70, followed by infection of HEK293T cells. We observed a similar 2-fold enhancement of infection and a proportional increase in spike incorporation as we did for D614G spike PVs (Figures S1A and S1B). Finally, to exclude the possibility that increased incorporation of S was specific for pseudotyped lentiviral particles, we generated coronavirus-like particles by co-transfection of WT or ΔH69/V70 S with SARS-CoV-2 membrane (M), envelope (E), and nucleocapsid (N) proteins, as described previously (Swann et al., 2020; Yurkovetskiy et al., 2020). Compared with levels of N, levels of cleaved S in coronavirus-like particles were again enhanced in the presence of the ΔH69/V70 (Figures S1C and S1D).

### Enhanced infectivity of ΔH69/V70 spike is not correlated with cleavage or entry route

**Figure 4:**
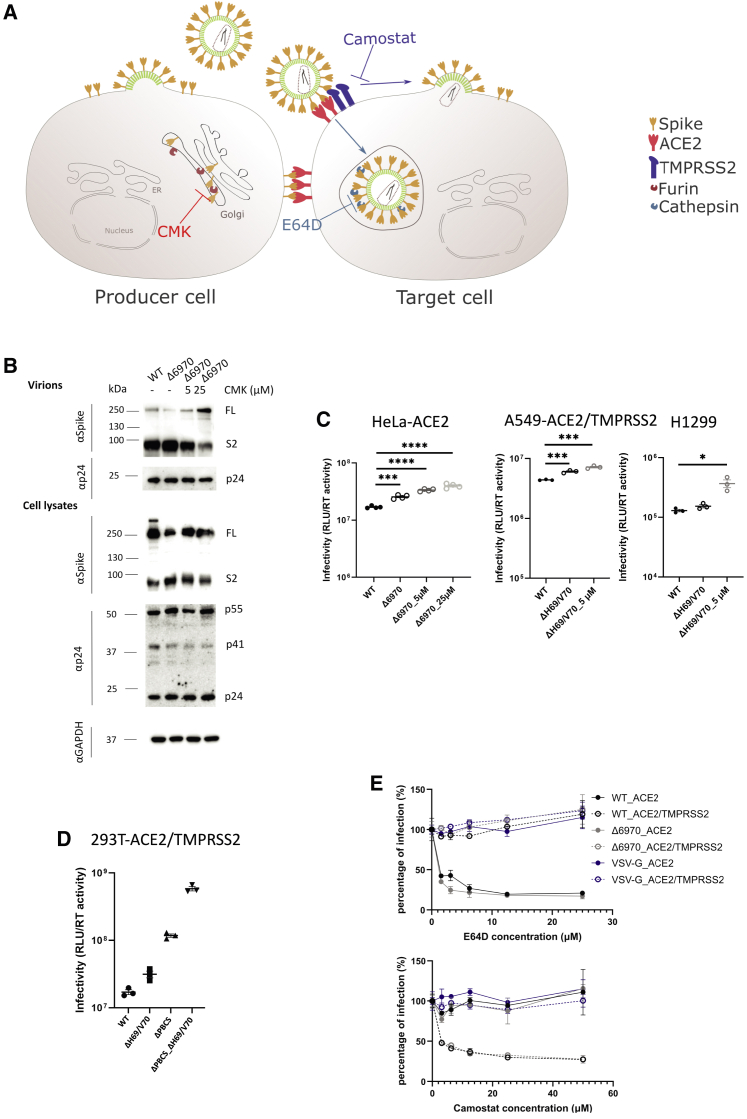
The route of SARS-CoV-2 S-mediated virus entry in cell lines is not altered by ΔH69/V70 spike (A) Schematic illustrating spike in producer cells with CMK targeting and blocking furin cleavage (left panel). In target cells, camostat inhibits TMPRSS2 and, therefore, cell fusion at the plasma membrane, and E64D blocks cathepsins and targets endocytic viral entry (right panel). (B) Western blots show that CMK inhibits spike S1/S2 cleavage in producer cells transfected with the S ΔH69/V70 plasmid, and spikes with altered S1/S2 cleavage are incorporated into the virions. Antibodies against HIV-1 p24 and spike S2 were used with anti-GAPDH as a loading control. (C) The viruses produced from transfected HEK293T cells in the presence of CMK were used to transduce target cells. The luciferase reading is used as a surrogate for the spike infectivity bearing with various S2/FL ratios. The data shown are technical triplicates or quadruplicates, and statical analysis was done using an unpaired t test. (D) Comparison of the infectivity of spike with the PBCS deleted (ΔPBCS) with and without ΔH69/V70. The effect of ΔH69/V70 is independent of the PBCS. (E) ΔH69/V70 does not alter the virus entry route. S pseudotyped lentiviruses bearing WT S, ΔH69/V70 S, or VSV-G were used to transduce 293T-ACE2 or 293T-ACE2/TMPRSS2 cells in the presence of E64D or camostat at different drug concentrations. The cells were then harvested after 2 days and assayed for luciferase expression, which was then normalized against the non-drug control (set as 100%). The data shown are technical duplicates. The data are representative of at least two independent experiments.

SARS-CoV-2 entry into target cells is thought to take place by two distinct routes following binding to ACE2 (Figure 4A). The first is an endosomal route where cathepsin is able to cleave spike with pH-dependent fusion in the endosome. The second route involves fusion at the plasma membrane with cleavage via the plasma membrane-associated protease TMPRSS2. To determine the mechanism by which increased spike cleavage in the context of ΔH69/V70 might affect entry, we used an inhibitor of furin cleavage (CMK) and protease inhibitors specific to the endosomal (ED64D) and plasma membrane fusion (camostat) entry routes (Figure 4A). We first treated producer cells with CMK and found that CMK inhibits spike S1/S2 cleavage in producer cells transfected with the S ΔH69/V70 plasmid and that the spikes with altered S1/S2 cleavage are incorporated into the virions (Figure 4B). We found that CMK treatment, although reducing S1/S2 cleavage, did not decrease PV infection in a variety of target cells (Figure 4C), suggesting that the increased infectivity in ΔH69/V70 is not due to more efficient cleavage of spike. To confirm our findings, we generated a spike lacking the polybasic cleavage site (PBCS) with or without ΔH69/V70 and tested PV infectivity on HEK293T cells overexpressing ACE2 and TMPRSS2. We found that deletion of the polybasic cleavage site led to increased infectivity of the PV, as observed previously for mutated PBCS (Papa et al., 2021). As expected, deletion of the PBCS did not alter the enhancing effect of ΔH69/V70 on PV infectivity (Figure 4D).

The altered level of S1/S2 cleavage in SARS-CoV-2 has been linked to its dependence on viral entry through membrane fusion or endocytosis in HEK293T and A549 cells (Peacock et al., 2020; Winstone et al., 2021). We therefore hypothesized that the increased spike cleavage of ΔH69/V70 S could influence the route of entry. To probe this, spike pseudotyped lentiviruses bearing WT spike, ΔH69/V70 spike, or vesicular stomatitis virus G protein (VSV-G) were used to transduce HEK293T-ACE2 or HEK293T-ACE2/TMPRSS2 cells in the presence of E64D or camostat at different drug concentrations (Figure 4E). As expected, the VSV-G pseudotyped particles were not affected by addition of E64D or camostat. Consistent with previous observations, the WT PV utilized endocytosis in the absence of TMPRSS2 (Peacock et al., 2020), but where TMPRSS2 was expressed, plasma membrane fusion became the dominant route (Papa et al., 2021; Winstone et al., 2021). However, there were no differences between WT and ΔH69/V70 in relative utilization of the endosomal versus plasma membrane entry routes. We conclude that the enhanced spike cleavage, although notable in ΔH69/V70, does not appear to be responsible for the increased infectivity of ΔH69/V70 spike observed in these cell line-based experiments.

### ΔH69/V70 spike compensates for reduced infectivity of RBD escape mutants

**Figure 5:**
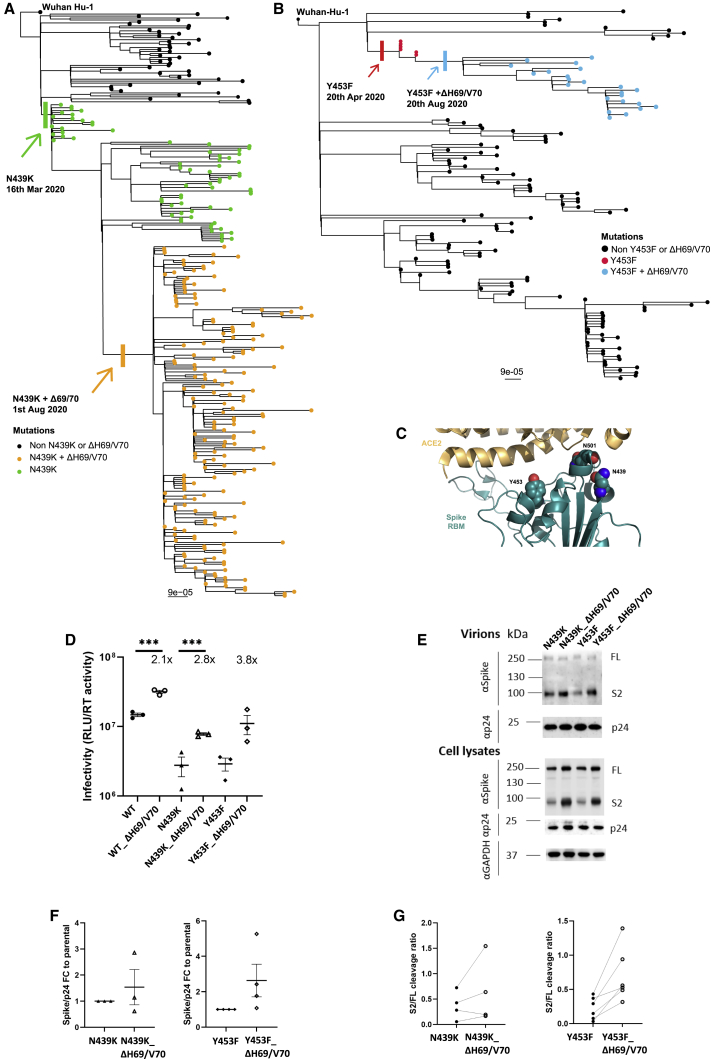
ΔH69/V70 appears after spike N439K and Y453F and compensates for their reduced infectivity (A and B) Maximum-likelihood phylogeny of global sequences carrying Spike mutant (A) N439K and (B) Y453F. All sequences in the GISAID database containing S:439K or S:Y453F (February 18, 2021) were downloaded, realigned to Wuhan-Hu-1 using MAFFT, and deduplicated. (C) Representation of the Spike RBM:ACE2 interface (PDB: 6M0J) with residues N439, Y453, and N501, highlighted as spheres colored by element. (D–F) Spike mutant ΔH69/V70 compensates for the infectivity defect of spike RBD mutations and is associated with increased spike incorporation into virions. (D) Infectivity of spike (D614G) ΔH69/V70 in the absence and presence of spike RBD mutations. Shown is single-round infection by luciferase-expressing lentiviruses pseudotyped with SARS-CoV-2 spike protein on target HeLa cells stably transduced with ACE2. Mean and SEM are shown. (E) Representative western blot of purified virions and cell lysates probed with antibodies against HIV-1 p24, SARS-CoV-2 spike S2, and GAPDH. (F and G) Densitometric quantification of the (F) spike:p24 and (G) cleaved S2 spike:FL spike ratios for spike (D614G) ΔH69/V70 in the absence and presence of spike RBD mutations across multiple experiments in pelleted viruses. U, unit of reverse transcriptase (RT) activity. Data are representative of at least two independent experiments. Student’s t test, ^∗∗∗^p < 0.001.

We next examined in greater detail the SARS-CoV-2 lineages where S mutations in the RBD were identified at high frequency and where ΔH69/V70 co-occurs. For example, N439K, an amino acid replacement reported to define variants increasing in numbers in Europe and other regions (Thomson et al., 2020; Figures 1 and 5A) now mostly co-occurs with ΔH69/V70. N439K appears to have reduced susceptibility to some convalescent sera as well as mAbs targeting the RBD while increasing affinity for ACE2 *in vitro* (Thomson et al., 2021). The first lineage possessing N439K (and not ΔH69/V70), B.1.141, is now extinct (Thomson et al., 2020). A second lineage with N439K, B.1.258, emerged later and subsequently acquired ΔH69/V70, leading to the initial rapid increase in the frequency of viruses possessing this deletion, spreading into Europe (Figure 1A; Brejová et al., 2021).

The second significant cluster with ΔH69/V70 and RBD mutants involves Y453F, another spike RBD mutation that increases binding affinity to ACE2 (Starr et al., 2020b), and has been found to be associated with mink-human infection (Munnink et al., 2021). Y453F has also been described as an escape mutation for mAb REGN10933, shows reduced susceptibility to convalescent sera (Baum et al., 2020; Hoffmann et al., 2021), and is possibly a T cell escape mutation (Motozono et al., 2021). ΔH69/V70 was first detected in the Y453F background on August 24, 2020 and so far appears to be limited to Danish sequences (Figures 1 and 5B), although an independent acquisition was recently reported along with ΔH69/V70 in an immune-compromised Russian individual with chronic infection (Bazykin et al., 2021).

We hypothesized that ΔH69/V70 might have arisen after Y453F and N439K to compensate for potential loss of infectivity, which has been reported previously for these RBD mutants (Motozono et al., 2021; Thomson et al., 2021). We therefore generated mutant spike plasmids bearing RBD mutations Y453F and N439K (Figure 5C) with and without ΔH69/V70 and performed infectivity assays in the lentiviral pseudotyping system. RBD mutations reduced infectivity of spike relative to the WT by around 2-fold (Figure 5D) and was partially rescued by ΔH69/V70. Based on observations of the effect of ΔH69/V70 on spike incorporation in the WT (Figure 3D), we predicted that the mechanism of increased infectivity for ΔH69/V70 in the context of RBD mutations might be similar. Analysis of virions from cell supernatants and cell lysates indeed showed an increased ratio of spike:p24 (Figures 5E and 5F). As observed for the WT, we also observed increased cleaved S2:FL when ΔH69/V70 was present along with the RBD mutants in PVs (Figures 5E and 5G).

### ΔH69/V70 is required for optimal B.1.1.7 Alpha variant spike S2 incorporation and infectivity

**Figure 6:**
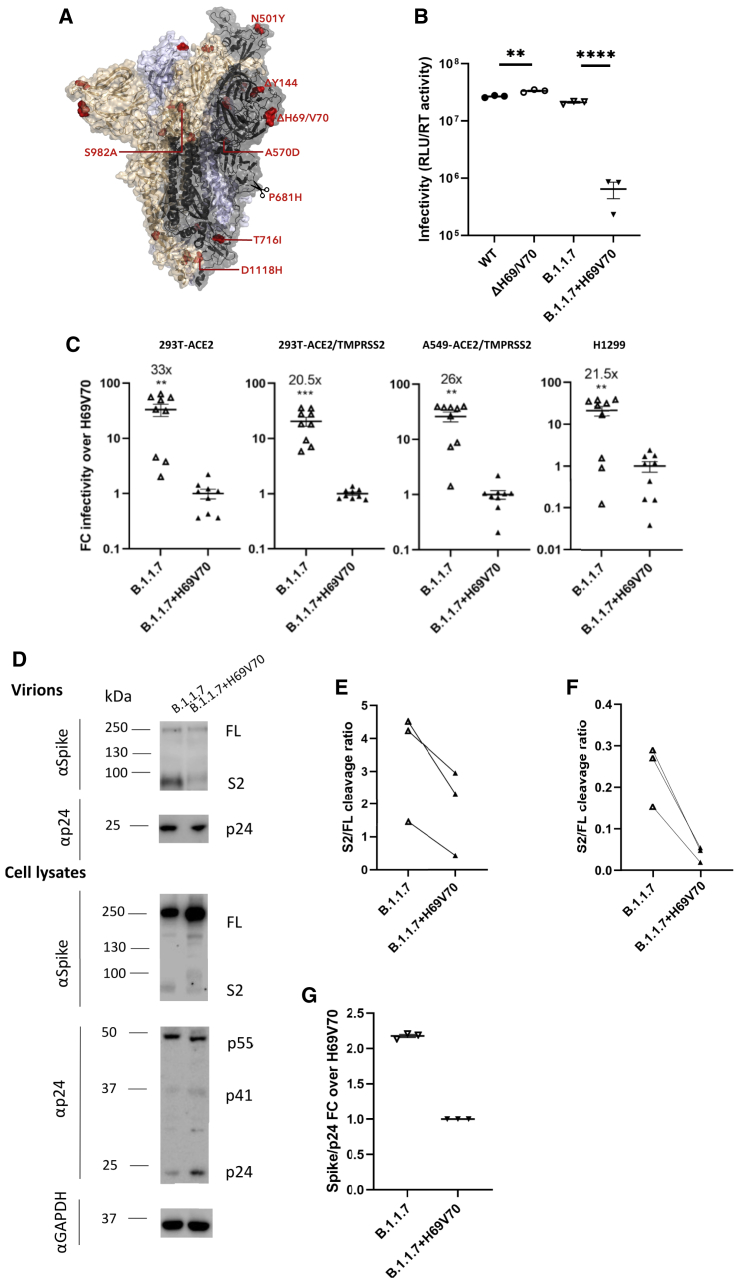
Spike ΔH69/V70 in B.1.1.7 enhances spike infectivity (A) Surface representation of the spike homotrimer in open conformation with one upright RBD overlaid with ribbon representation (PDB: 6ZGG; Wrobel et al., 2020), with different monomers shown in black, pale blue, and gold. The deleted residues H69 and V70 and the residues involved in amino acid substitutions (501, 570, 716, 982, and 1118) and the deletion at position 144 are colored red on each monomer and labeled on the monomer with an upright RBD shown in black. Scissors mark the approximate location of an exposed loop (residues 677–688) containing the furin cleavage site and including residue 681, which is absent from the structure. (B) Representative infectivity of B.1.1.7 with replacement of H69 and V70 versus B.1.1.7 containing spike ΔH69/V70 and WT (D614G) spike; single-round infection by luciferase-expressing lentiviruses pseudotyped with SARS-CoV-2 spike protein on HeLa cells transduced with ACE2. The data represent technical triplicates. (C) Fold change of luciferase expression over the replacement of H69V70 in ACE2-transfected and ACE2- and TMPRSS2-transfected HEK293T cells, A459-ACE2/TMPRSS2 cells, and H1299 cells. The data shown are from three independent experiments, each in technical triplicates (one-sample t test). (D) Representative western blot analysis following transfection of HEK293T cells with spike and lentiviral plasmids. Virion loading was normalized for input virus using RT activity. Antibodies against HIV-1 p24 and spike S2 were used with anti-GAPDH as a loading control. (E and F) S2 to FL spike was analyzed by densitometry, and the S2:FL cleavage ratio was calculated for virions (E) and cell lysates (F). (G) Quantification of the spike:p24 ratio for B.1.1.7 and B.1.1.7 with H69/V70 replacement across three independent experiments. ^∗∗^p < 0.01, ^∗∗∗∗^p < 0.0001.

A lineage containing ΔH69/V70 was first detected in the United Kingdom with the RBD mutation N501Y along with multiple other spike and other mutations (Figure 1; Figure S2). Sequences belonging to this lineage were subsequently called B.1.1.7 and classified as a variant of concern (VOC) due to a higher rate of transmission (Volz et al., 2021b). It was more recently named the Alpha variant by WHO. Subsequently B.1.1.7 has spread rapidly to over 100 countries, exemplifying a new chapter in the pandemic, with additional VOCs detected in other geographical locations. In addition to RBD N501Y and NTD ΔH69/V70, B.1.1.7 is defined by further S mutations across S2 (T716I, S982A, and D1118H) and S1 (ΔY144, A570D, and P681H) (Figure 6A; Rambaut et al., 2020). The available sequence data did not enable determination of whether the Alpha variant B.1.1.7 mutations N501Y and ΔH69/V70 arose as a result of an N501Y virus acquiring ΔH69/V70 or vice versa, although a United Kingdom Alpha variant B.1.1.7 sequence was identified with N501Y, A570D, ΔH69/V70, and D1118H (Figure S2).

To ascertain whether H69V70 is a target for neutralizing antibodies in the context of the Alpha variant B.1.1.7, we first tested 12 NTD-specific mAbs isolated from 4 individuals who recovered from WT SARS-CoV-2 infection with an *in-vitro* PV neutralization assay using WT SARS-CoV-2 S and the B.1.1.7 S or B.1.1.7 S with reversion of the H69/V70 deletions (B.1.1.7 H69/V70) PVs in VeroE6 target cells expressing TMPRSS2. We found that 7 of 12 NTD-specific mAbs (58%) showed a marked decrease or complete loss of neutralizing activity to B.1.1.7 and B.1.1.7 H69/V70 (>30-fold-change reduction), suggesting that, in a sizeable fraction of NTD antibodies, the H69/V70 deletion is not responsible for their loss of neutralizing activity (Figure S3). The remaining 5 mAbs showed a partial reduction (2- to 10-fold) in Alpha variant B.1.1.7 neutralization that was not rescued by reversion of the H69/V70 deletions.

Given our data on introduction of ΔH69/V70 into the WT (Figure 3), we hypothesized that ΔH69/V70 was selected in the evolution of Alpha variant B.1.1.7 to increase viral entry. We predicted that replacement of H69 and V70 would impair the infectivity of the B.1.1.7 PV and reduce total spike levels. To examine this, we compared the infectivity of the B.1.1.7 spike PV versus the B.1.1.7 PV with restored H69 and V70. We observed that B.1.1.7 infectivity was slightly lower than that of the WT (Figure 6B). As expected, we observed a significant reduction in infectivity for viruses where H69 and V70 had been re-inserted across a number of cell types, including H1299 expressing endogenous levels of ACE2 and TMPRSS2 receptors (Figures 6B and 6C). When we measured spike incorporation into virions, we found that the reduced infectivity of B.1.1.7 with replaced H69 V70 was associated with reduced spike:p24 and S2:FL ratios, as expected (Figures 6D–6G).

### Alpha variant B.1.1.7 spike mediates faster syncytium formation and is ΔH69/V70 dependent

**Figure 7:**
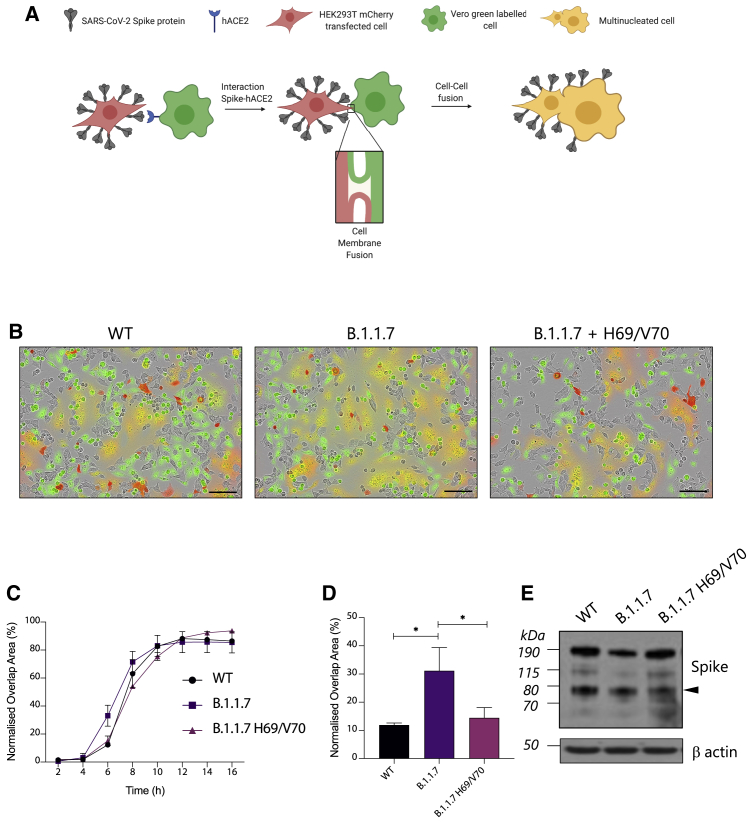
ΔH69/V70 significantly accelerates cell-cell fusion activity of Alpha variant B.1.1.7 spike protein (A) Schematic of the cell-cell fusion assay (created with BioRender). (B) Reconstructed images at 6 h of HEK293T cells co-transfected with the indicated spike mutants and mCherry-expressing plasmid mixed with green dye-labeled Vero acceptor cells. Scale bars represent 100 mm. Green identifies acceptor cells, and red marks donor cells. Merged green-red indicates syncytia. (C) Quantification of cell-cell fusion kinetics, showing percentage of green and red overlap area over time. Mean is plotted, with error bars representing SEM. (D) Quantification of cell-cell fusion of the indicated spike mutants 6 h after transfection. Mean is plotted, with error bars representing SEM. (E) Representative western blot of cells transfected with the indicated spike mutants (detected with anti-S2 antibody). The S2 subunit is indicated by an arrowhead. β-Actin is shown as a loading control. Data are representative of at least three independent experiments. ^∗^p < 0.05, unpaired t test.

Previous reports have shown that the SARS-CoV-2 spike protein localizes to the cell host plasma membrane and possesses high fusogenic activity, triggering formation of large multi-nucleated cells (called syncytia) *in vitro* and *in vivo*, potentially providing an additional and a more rapid route for virus dissemination among neighbor cells (Bussani et al., 2020; Cattin-Ortolá et al., 2020; Papa et al., 2021). The role of syncytium formation in viral replication and pathogenesis of severe coronavirus disease 2019 (COVID-19) has been reported and may be a druggable process to treat COVID-19 pathology (Braga et al., 2021). We expressed B.1.1.7 spike or a B.1.1.7 with restored H69 and V70 together with the mCherry fluorescent protein in HEK293T cells and labeled Vero cells with a green fluorescent dye (Figure 7A). All spike constructs showed similar protein expression and achieved similar cell-cell fusion by 16 h. B.1.1.7 appeared to mediate more cell-cell fusion events over earlier time points, with the color overlap area being 2–3 times greater for B.1.1.7 compared with the WT 6 h after mixing. Interestingly, this enhancement was abrogated by re-insertion of the H69 and V70 residues (Figures 7B–7D). We conclude that B.1.1.7 spike mediates faster fusion kinetics than the WT bearing D614G Wuhan-1 spike that is dependent on ΔH69/V70.

### ΔH69/V70 does not enhance infectivity of bat coronavirus RaTG13 spike

Finally, to investigate the importance of this part of spike beyond SARS-CoV-2 for other coronaviruses with zoonotic potential, we examined the 69/70 region of spike in a set of other known sarbecoviruses (Figures S4A–S4C). We observed substantial variability in the region, resulting in frequent insertions or deletions (indels), with some viruses including SARS-CoV having 6- to 7-amino-acid deletions (Figure S4B). This is indicative of high structural plasticity in this protein region that could allow sarbecoviruses to alter their spike conformation. RaTG13 is the evolutionarily closest relative to SARS-CoV-2 for this region and, after RaTG13, is the cluster of 5 CoVs sampled in trafficked pangolins in the Guangxi province (Lam et al., 2020). Inspection of the 69/70 region in these virus sequences raises the interesting observation that one of the five viruses in the cluster, P2V, has amino acids 69H and 70L present, whereas the other four have a double amino acid deletion (Figures S4A–S4C). Given that SARS-CoV-2 and RaTG13 have the homologous HV insertion at these positions, one explanation is that the proximal common ancestor between SARS-CoV-2 and the Guangxi pangolin cluster had the insertion, which was then lost while circulating in the pangolin population, similar to observations with SARS-CoV-2 in humans. However, the fact that P2V was cultured in Vero E6 cells prior to sequencing (contrary to the other 4, sequenced directly from the pangolin sample) raises the possibility of this being an independent insertion, favored as a monkey cell line-specific adaptation. Interestingly, the double amino acid indel in the pangolin viruses is in frame, in contrast to SARS-CoV-2.

Furthermore, the two almost identical bat viruses sequenced recently from Cambodian samples, RShSTT182 and RShSTT200 (Hul et al., 2021), possess an H69V70 insertion despite being more distantly related to SARS-CoV-2 for this region of spike (Figures S4A–S4C). This independent occurrence of the insertion is suggestive of context-dependent selective pressure playing a role in recurring gain and loss of these two residues in sarbecoviruses. To test whether the fitness effect associated with acquisition of ΔH69/V70 is specific to SARS-CoV-2 and not other sarbecovirus spike backgrounds, we cloned FL S from RaTG13 and generated pseudotyped lentiviruses expressing RaTG13 spike protein as well as a RaTG13 S ΔH69/V70 counterpart. We observed that cleaved and uncleaved S expression levels in the PV did not differ between WT and the ΔH69/V70 RaTG13 spike and that there was no difference in infectivity on target cells expressing ACE2 or ACE2 and TMPRSS2 (Figures S4D and S4E). This result suggests that, as one would expect, the enhancing effect of ΔH69/V70 on spike levels and infectivity is specific to the spike background.

## Discussion

We have presented data demonstrating multiple independent and circulating lineages of SARS-CoV-2 variants bearing spike ΔH69/V70. This recurring deletion, spanning 6 nt, is due to an out-of-frame deletion of 6 nt and occurs in the terminal loop of a helix loop motif within the predicted RNA structure, as do other NTD deletions observed in new variants, such as B.1.1.7 (Alpha), B.1.617 (Delta), B.1.351 (Beta), and P.1 (Gamma) (Figure S5). Stable helix loop motifs are associated with pausing/dissociation events in reverse transcriptase (Harrison et al., 1998). Because all nucleic acid polymerases have a common ancestor with homologous dinucleotide triphosphate (dNTP) binding motifs and similar global structures (Delarue et al., 1990; Ollis et al., 1985; Sousa et al., 1993), it is probable that all RNA polymerases use similar mechanisms for transcript termination (Reeder and Lang, 1994). A recent in-cell biochemical analysis of SARS-CoV2 RNA structure showed nucleotide reactivity consistent with this model within these stem-loops (Huston et al., 2021). These analyses provide a rationale for preferential emergence of ΔH69/V70 and other deletions, such as the well-described NTD antibody escape deletion ΔY144 (Chi et al., 2020; McCarthy et al., 2020, 2021) (in B.1.1.7 and the recently reported B.1.525) at the terminal loops of helical loop motifs. ΔH69/V70 itself has frequently followed immune escape-associated amino acid replacements in the RBD (e.g., N439K and Y453F) and is specifically found in the B.1.1.7 variant, known to have higher transmissibility (Volz et al., 2021a) and, possibly, pathogenicity (Davies et al., 2021).

We find that ΔH69/V70 does not significantly reduce the sensitivity of spike to neutralizing antibodies in serum from a group of recovered individuals or binding of multiple mAbs directed against the NTD. In addition, we have shown that repair of ΔH69/V70 does not appreciably alter the potency of NTD antibodies against the B.1.1.7 spike. Thus, the deletion is unlikely to be an immune escape mechanism. Instead, our experimental results demonstrate that ΔH69/V70 is able increase infectivity of the Wuhan-1 D614G spike PV as well as the PV bearing the additional RBD mutations N439K or Y453F, explaining why the deletion is often observed after these immune escape mutations that carry infectivity cost (Motozono et al., 2021; Thomson et al., 2021). We show that the mechanism of enhanced infectivity across the RBD mutations tested is associated with greater spike incorporation into virions where ΔH69/V70 is present. The phenotype is also independent of producer cells used. Importantly, we were able to recapitulate the ΔH69/V70 phenotype in a spike protein that did not have the D614G mutation, indicating that D614G is not involved in the mechanism. These observations are supported by ΔH69/V70 being observed in D614 viruses in January 2020 in the United States and Thailand. Although we did not use a replication-competent system, a recent study reports that the ΔH69/V70 mutated Washington strain virus isolate confers increased replication in cell lines and higher viral loads in hamsters (Liu et al., 2021). Indeed, ΔH69 has been observed in cell culture during remdesivir selection experiments with a replication-competent virus, consistent with a replication advantage (Szemiel et al., 2021).

We have found consistent differences in spike as well as cleaved spike in the producer cell and its incorporation into PV particles when comparing ΔH69/V70 with a Wuhan-1 spike (both with D614G). This could be explained by stability during intracellular trafficking or the route taken to the surface, differences in post-translational modification of the spike protein, or membrane characteristics at the budding site of virus or virus-like particles. Because the amount of spike incorporation into virions reflects the spike in the cells, virion formation is likely unaffected by ΔH69/V70. Interestingly, although pharmacological inhibition of furin by CMK in producer cells did prevent S1/S2 cleavage and altered the balance of S2:FL spike in cells and virions, PV infectivity was not reduced by drug treatment. These data suggest that the increase in entry efficiency conferred by spike ΔH69/V70 is independent of spike S1/S2 cleavage. Similar findings regarding the lack of relationship between the balance of S2:FL and infectivity have been reported in the context of furin knockout cells rather than furin inhibition with CMK (Papa et al., 2021). In addition, a recent report on the S1/S2 cleavage site mutation P681H demonstrated that enhanced cleavage of spike P681H was not associated with increased PV infectivity or cell fusion relative to the WT (Lubinski et al., 2021). There may, however, be differences *in vivo*.

We explored the entry route of ΔH69/V70 spike using cathepsin inhibition to block endosomal entry and camostat to block entry via plasma membrane fusion. ΔH69/V70 spike was as sensitive to camostat and the cathepsin inhibitor ED64 as the WT, arguing that the efficiency of entry route is similar despite differences in cleaved spike. Although S1/S2 cleavage allows avoidance of endosome-associated IFITM restriction and appears to be critical for transmission in animal models (Peacock et al., 2020), cleaved spike may be less infectious when S1 is shed prematurely, possibly conferring a disadvantage under some circumstances. In addition, Peacock et al. (2020) showed that S1/S2 cleavage in the producer cell, conferred by a polybasic stretch at the cleavage site, is advantageous in cells expressing abundant TMPRSS2 but deleterious in cells lacking TMPRSS2.

The Alpha variant Al (B.1.1.7), bearing seven spike mutations, is responsible for a new pandemic phase that is demonstrably more pathogenic (Davies et al., 2021) and more transmissible (Volz et al., 2021a). Detection of a high number of novel mutations suggests that this lineage has been introduced from a geographic region with very poor sampling or that viral evolution may have occurred in a single individual in the context of chronic infection (Kemp et al., 2021).

We show that Alpha variant B.1.1.7 spike has similar infectivity as WT D614G spike, consistent with data on live B.1.1.7 virus in human airway epithelial cells (Brown et al., 2021) but in contrast to another study that showed a difference in live virus with the 8 spike mutations (Liu et al., 2021). Importantly, however, we demonstrate loss of infectivity when the H69/V70 amino acids are replaced in B.1.1.7 S, accompanied, as expected, by reduced S1/S2 cleavage and reduced S incorporation into virions. These data point to epistatic interactions between observed mutations in spike of B.1.1.7 with a trade-off between mutations that incur virus entry cost with those that contribute to other activities, such as immune evasion.

Of greatest potential importance is our observation that Alpha variant B.1.1.7 spike mediates faster syncytium formation and that this enhanced cell-cell fusion activity is dependent on ΔH69/V70. Syncytium formation is a key feature of severe and fatal COVID-19 (Bussani et al., 2020) and implicated in elevated viral replication (Braga et al., 2021). We speculate that the increased fusogenicity of B.1.1.7 spike may contribute to the higher mortality (Davies et al., 2021) and transmissibility of B.1.1.7 (Volz et al., 2021a).

### Limitations

Although we combined epidemiological, evolutionary, protein and RNA structure, and experimental data in our study, a limitation is that the experiments were conducted with PVs and coronavirus-like particles rather than replication-competent viruses. We also carried out experiments in cells overexpressing receptors, although the results were recapitulated in lung cell lines expressing endogenous levels of ACE2 and TMPRSS2.

Detection and surveillance of B.1.1.7 has been facilitated in the United Kingdom by the phenomenon of SGTF (S[pike] gene target failure) because of primers in the Thermo Fisher Scientific SARS-CoV-2 diagnostic qPCR assay used by a significant number of testing facilities. The S gene target (binding in the region of H69/V70) is one of three; therefore, a marker for the spread of B.1.1.7 has been tracked by loss of signal in the S gene target (Volz et al., 2021b). However, recent reports from the United States and central Europe caution against use of SGTF as a sole marker for B.1.1.7 detection because a significant ΔH69/V70 lineage without other mutations in spike is circulating in the United States, and a B.1.258 lineage with N439K with ΔH69/V70 is circulating in Slovakia/Czech Republic (Brejová et al., 2021; Larsen and Worobey, 2020). Such examples highlight the need for genome sequencing to accompany novel approaches to diagnostics for variants.

Given the emergence of multiple clusters of variants carrying RBD mutations and ΔH69/V70 , limitation of transmission takes on renewed urgency. As another example, a new VOC bearing ΔH69/V70 with E484K was recently identified (B.1.525). Comprehensive vaccination efforts should be accelerated to limit transmission and acquisition of further mutations, and future vaccines could include ΔH69/V70 to close this route for virus evolution, assuming that effective neutralizing antibodies to this region are generated. Fortunately, our experiments with RaTG13 demonstrate that ΔH69/V70 may not enhance the infectivity of other bat sarbecoviruses with zoonotic potential.

We found that a two-amino-acid deletion, ΔH69/V70, promotes SARS-CoV-2 spike incorporation into viral particles and increases infectivity by a mechanism that remains to be fully explained. This deletion has arisen multiple times and often after spike antibody escape mutations that reduce spike-mediated entry efficiency. Critically, B.1.1.7 spike mediates faster syncitium formation, and this enhanced cell-cell fusion activity is dependent on ΔH69/V70. In addition, B.1.1.7 spike requires ΔH69/V70 for optimal infectivity, and we conclude that ΔH69/V70 enables SARS-CoV-2 to tolerate multiple immune escape mutations while maintaining infectivity and fusogenicity.

## Consortia

The members of the COVID-19 Genomics UK (COG-UK) Consortium are Samuel C. Robson, Nicholas J. Loman, Thomas R. Connor, Tanya Golubchik, Rocio T. Martinez Nunez, Catherine Ludden, Sally Corden, Ian Johnston, David Bonsall, Colin P. Smith, Ali R. Awan, Giselda Bucca, M. Estee Torok, Kordo Saeed, Jacqui A. Prieto, David K. Jackson, William L. Hamilton, Luke B. Snell, Catherine Moore, Ewan M. Harrison, Sonia Goncalves, Derek J. Fairley, Matthew W. Loose, Joanne Watkins, Rich Livett, Samuel Moses, Roberto Amato, Sam Nicholls, Matthew Bull, Darren L. Smith, Jeff Barrett, David M. Aanensen, Martin D. Curran, Surendra Parmar, Dinesh Aggarwal, James G. Shepherd, Matthew D. Parker, Sharon Glaysher, Matthew Bashton, Anthony P. Underwood, Nicole Pacchiarini, Katie F. Loveson, Kate E. Templeton, Cordelia F. Langford, John Sillitoe, Thushan I. de Silva, Dennis Wang, Dominic Kwiatkowski, Andrew Rambaut, Justin O’Grady, Simon Cottrell, Matthew T. G. Holden, Emma C. Thomson, Husam Osman, Monique Andersson, Anoop J. Chauhan, Mohammed O. Hassan-Ibrahim, Mara Lawniczak, Alex Alderton, Meera Chand, Chrystala Constantinidou, Meera Unnikrishnan, Alistair C. Darby, Julian A. Hiscox, Steve Paterson, Inigo Martincorena, Erik M. Volz, Andrew J. Page, Oliver G. Pybus, Andrew R. Bassett, Cristina V. Ariani, Michael H. Spencer Chapman, Kathy K. Li, Rajiv N. Shah, Natasha G. Jesudason, Yusri Taha, Martin P. McHugh, Rebecca Dewar, Aminu S. Jahun, Claire McMurray, Sarojini Pandey, James P. McKenna, Andrew Nelson, Gregory R. Young, Clare M. McCann, Scott Elliott, Hannah Lowe, Ben Temperton, Sunando Roy, Anna Price, Sara Rey, Matthew Wyles, Stefan Rooke, Sharif Shaaban, Mariateresa de Cesare, Laura Letchford, Siona Silveira, Emanuela Pelosi, Eleri Wilson-Davies, Myra Hosmillo, Áine O’Toole, Andrew R. Hesketh, Richard Stark, Louis du Plessis, Chris Ruis, Helen Adams, Yann Bourgeois, Stephen L. Michell, Dimitris Gramatopoulos, Jonathan Edgeworth, Judith Breuer, John A. Todd, Christophe Fraser, David Buck, Michaela John, Gemma L. Kay, Steve Palmer, Sharon J. Peacock, David Heyburn, Danni Weldon, Esther Robinson, Alan McNally, Peter Muir, Ian B. Vipond, John Boyes, Venkat Sivaprakasam, Tranprit Salluja, Samir Dervisevic, Emma J. Meader, Naomi R. Park, Karen Oliver, Aaron R. Jeffries, Sascha Ott, Ana da Silva Filipe, David A. Simpson, Chris Williams, Jane A. H. Masoli, Bridget A. Knight, Christopher R. Jones, Cherian Koshy, Amy Ash, Anna Casey, Andrew Bosworth, Liz Ratcliffe, Li Xu-McCrae, Hannah M. Pymont, Stephanie Hutchings, Lisa Berry, Katie Jones, Fenella Halstead, Thomas Davis, Christopher Holmes, Miren Iturriza-Gomara, Anita O. Lucaci, Paul Anthony Randell, Alison Cox, Pinglawathee Madona, Kathryn Ann Harris, Julianne Rose Brown, Tabitha W. Mahungu, Dianne Irish-Tavares, Tanzina Haque, Jennifer Hart, Eric Witele, Melisa Louise Fenton, Steven Liggett, Clive Graham, Emma Swindells, Jennifer Collins, Gary Eltringham, Sharon Campbell, Patrick C. McClure, Gemma Clark, Tim J. Sloan, Carl Jones, Jessica Lynch, Ben Warne, Steven Leonard, Jillian Durham, Thomas Williams, Sam T. Haldenby, Nathaniel Storey, Nabil-Fareed Alikhan, Nadine Holmes, Christopher Moore, Matthew Carlile, Malorie Perry, Noel Craine, Ronan A. Lyons, Angela H. Beckett, Salman Goudarzi, Christopher Fearn, Kate Cook, Hannah Dent, Hannah Paul, Robert Davies, Beth Blane, Sophia T. Girgis, Mathew A. Beale, Katherine L. Bellis, Matthew J. Dorman, Eleanor Drury, Leanne Kane, Sally Kay, Samantha McGuigan, Rachel Nelson, Liam Prestwood, Shavanthi Rajatileka, Rahul Batra, Rachel J. Williams, Mark Kristiansen, Angie Green, Anita Justice, Adhyana I. K. Mahanama, Buddhini Samaraweera, Nazreen F. Hadjirin, Joshua Quick, Radoslaw Poplawski, Leanne M. Kermack, Nicola Reynolds, Grant Hall, Yasmin Chaudhry, Malte L. Pinckert, Iliana Georgana, Robin J. Moll, Alicia Thornton, Richard Myers, Joanne Stockton, Charlotte A. Williams, Wen C. Yew, Alexander J. Trotter, Amy Trebes, George MacIntyre-Cockett, Alec Birchley, Alexander Adams, Amy Plimmer, Bree Gatica-Wilcox, Caoimhe McKerr, Ember Hilvers, Hannah Jones, Hibo Asad, Jason Coombes, Johnathan M. Evans, Laia Fina, Lauren Gilbert, Lee Graham, Michelle Cronin, Sara Kumziene-Summerhayes, Sarah Taylor, Sophie Jones, Danielle C. Groves, Peijun Zhang, Marta Gallis, Stavroula F. Louka, Igor Starinskij, Chris Jackson, Marina Gourtovaia, Gerry Tonkin-Hill, Kevin Lewis, Jaime M. Tovar-Corona, Keith James, Laura Baxter, Mohammad T. Alam, Richard J. Orton, Joseph Hughes, Sreenu Vattipally, Manon Ragonnet-Cronin, Fabricia F. Nascimento, David Jorgensen, Olivia Boyd, Lily Geidelberg, Alex E. Zarebski, Jayna Raghwani, Moritz U. G. Kraemer, Joel Southgate, Benjamin B. Lindsey, Timothy M. Freeman, Jon-Paul Keatley, Joshua B. Singer, Leonardo de Oliveira Martins, Corin A. Yeats, Khalil Abudahab, Ben E. W. Taylor, Mirko Menegazzo, John Danesh, Wendy Hogsden, Sahar Eldirdiri, Anita Kenyon, Jenifer Mason, Trevor I. Robinson, Alison Holmes, James Price, John A. Hartley, Tanya Curran, Alison E. Mather, Giri Shankar, Rachel Jones, Robin Howe, Sian Morgan, Elizabeth Wastenge, Michael R. Chapman, Siddharth Mookerjee, Rachael Stanley, Wendy Smith, Timothy Peto, David Eyre, Derrick Crook, Gabrielle Vernet, Christine Kitchen, Huw Gulliver, Ian Merrick, Martyn Guest, Robert Munn, Declan T. Bradley, Tim Wyatt, Charlotte Beaver, Luke Foulser, Sophie Palmer, Carol M. Churcher, Ellena Brooks, Kim S. Smith, Katerina Galai, Georgina M. McManus, Frances Bolt, Francesc Coll, Lizzie Meadows, Stephen W. Attwood, Alisha Davies, Elen De Lacy, Fatima Downing, Sue Edwards, Garry P. Scarlett, Sarah Jeremiah, Nikki Smith, Danielle Leek, Sushmita Sridhar, Sally Forrest, Claire Cormie, Harmeet K. Gill, Joana Dias, Ellen E. Higginson, Mailis Maes, Jamie Young, Michelle Wantoch, Dorota Jamrozy, Stephanie Lo, Minal Patel, Verity Hill, Claire M. Bewshea, Sian Ellard, Cressida Auckland, Ian Harrison, Chloe Bishop, Vicki Chalker, Alex Richter, Andrew Beggs, Angus Best, Benita Percival, Jeremy Mirza, Oliver Megram, Megan Mayhew, Liam Crawford, Fiona Ashcroft, Emma Moles-Garcia, Nicola Cumley, Richard Hopes, Patawee Asamaphan, Marc O. Niebel, Rory N. Gunson, Amanda Bradley, Alasdair Maclean, Guy Mollett, Rachel Blacow, Paul Bird, Thomas Helmer, Karlie Fallon, Julian Tang, Antony D. Hale, Louissa R. Macfarlane-Smith, Katherine L. Harper, Holli Carden, Nicholas W. Machin, Kathryn A. Jackson, Shazaad S. Y. Ahmad, Ryan P. George, Lance Turtle, Elaine O’Toole, Joanne Watts, Cassie Breen, Angela Cowell, Adela Alcolea-Medina, Themoula Charalampous, Amita Patel, Lisa J. Levett, Judith Heaney, Aileen Rowan, Graham P. Taylor, Divya Shah, Laura Atkinson, Jack C. D. Lee, Adam P. Westhorpe, Riaz Jannoo, Helen L. Lowe, Angeliki Karamani, Leah Ensell, Wendy Chatterton, Monika Pusok, Ashok Dadrah, Amanda Symmonds, Graciela Sluga, Zoltan Molnar, Paul Baker, Stephen Bonner, Sarah Essex, Edward Barton, Debra Padgett, Garren Scott, Jane Greenaway, Brendan A. I. Payne, Shirelle Burton-Fanning, Sheila Waugh, Veena Raviprakash, Nicola Sheriff, Victoria Blakey, Lesley-Anne Williams, Jonathan Moore, Susanne Stonehouse, Louise Smith, Rose K. Davidson, Luke Bedford, Lindsay Coupland, Victoria Wright, Joseph G. Chappell, Theocharis Tsoleridis, Jonathan Ball, Manjinder Khakh, Vicki M. Fleming, Michelle M. Lister, Hannah C. Howson-Wells, Louise Berry, Tim Boswell, Amelia Joseph, Iona Willingham, Nichola Duckworth, Sarah Walsh, Emma Wise, Nathan Moore, Matilde Mori, Nick Cortes, Stephen Kidd, Rebecca Williams, Laura Gifford, Kelly Bicknell, Sarah Wyllie, Allyson Lloyd, Robert Impey, Cassandra S. Malone, Benjamin J. Cogger, Nick Levene, Lynn Monaghan, Alexander J. Keeley, David G. Partridge, Mohammad Raza, Cariad Evans, Kate Johnson, Emma Betteridge, Ben W. Farr, Scott Goodwin, Michael A. Quail, Carol Scott, Lesley Shirley, Scott A. J. Thurston, Diana Rajan, Iraad F. Bronner, Louise Aigrain, Nicholas M. Redshaw, Stefanie V. Lensing, Shane McCarthy, Alex Makunin, Carlos E. Balcazar, Michael D. Gallagher, Kathleen A. Williamson, Thomas D. Stanton, Michelle L. Michelsen, Joanna Warwick-Dugdale, Robin Manley, Audrey Farbos, James W. Harrison, Christine M. Sambles, David J. Studholme, Angie Lackenby, Tamyo Mbisa, Steven Platt, Shahjahan Miah, David Bibby, Carmen Manso, Jonathan Hubb, Gavin Dabrera, Mary Ramsay, Daniel Bradshaw, Ulf Schaefer, Natalie Groves, Eileen Gallagher, David Lee, David Williams, Nicholas Ellaby, Hassan Hartman, Nikos Manesis, Vineet Patel, Juan Ledesma, Katherine A. Twohig, Elias Allara, Clare Pearson, Jeffrey K. J. Cheng, Hannah E. Bridgewater, Lucy R. Frost, Grace Taylor-Joyce, Paul E. Brown, Lily Tong, Alice Broos, Daniel Mair, Jenna Nichols, Stephen N. Carmichael, Katherine L. Smollett, Kyriaki Nomikou, Elihu Aranday-Cortes, Natasha Johnson, Seema Nickbakhsh, Edith E. Vamos, Margaret Hughes, Lucille Rainbow, Richard Eccles, Charlotte Nelson, Mark Whitehead, Richard Gregory, Matthew Gemmell, Claudia Wierzbicki, Hermione J. Webster, Chloe L. Fisher, Adrian W. Signell, Gilberto Betancor, Harry D. Wilson, Gaia Nebbia, Flavia Flaviani, Alberto C. Cerda, Tammy V. Merrill, Rebekah E. Wilson, Marius Cotic, Nadua Bayzid, Thomas Thompson, Erwan Acheson, Steven Rushton, Sarah O’Brien, David J. Baker, Steven Rudder, Alp Aydin, Fei Sang, Johnny Debebe, Sarah Francois, Tetyana I. Vasylyeva, Marina Escalera Zamudio, Bernardo Gutierrez, Angela Marchbank, Joshua Maksimovic, Karla Spellman, Kathryn McCluggage, Mari Morgan, Robert Beer, Safiah Afifi, Trudy Workman, William Fuller, Catherine Bresner, Adrienn Angyal, Luke R. Green, Paul J. Parsons, Rachel M. Tucker, Rebecca Brown, Max Whiteley, James Bonfield, Christoph Puethe, Andrew Whitwham, Jennifier Liddle, Will Rowe, Igor Siveroni, Thanh Le-Viet, Amy Gaskin, Rob Johnson, Irina Abnizova, Mozam Ali, Laura Allen, Ralph Anderson, Cristina Ariani, Siobhan Austin-Guest, Sendu Bala, Jeffrey Barrett, Andrew Bassett, Kristina Battleday, James Beal, Mathew Beale, Sam Bellany, Tristram Bellerby, Katie Bellis, Duncan Berger, Matt Berriman, Paul Bevan, Simon Binley, Jason Bishop, Kirsty Blackburn, Nick Boughton, Sam Bowker, Timothy Brendler-Spaeth, Iraad Bronner, Tanya Brooklyn, Sarah Kay Buddenborg, Robert Bush, Catarina Caetano, Alex Cagan, Nicola Carter, Joanna Cartwright, Tiago Carvalho Monteiro, Liz Chapman, Tracey-Jane Chillingworth, Peter Clapham, Richard Clark, Adrian Clarke, Catriona Clarke, Daryl Cole, Elizabeth Cook, Maria Coppola, Linda Cornell, Clare Cornwell, Craig Corton, Abby Crackett, Alison Cranage, Harriet Craven, Sarah Craw, Mark Crawford, Tim Cutts, Monika Dabrowska, Matt Davies, Joseph Dawson, Callum Day, Aiden Densem, Thomas Dibling, Cat Dockree, David Dodd, Sunil Dogga, Matthew Dorman, Gordon Dougan, Martin Dougherty, Alexander Dove, Lucy Drummond, Monika Dudek, Laura Durrant, Elizabeth Easthope, Sabine Eckert, Pete Ellis, Ben Farr, Michael Fenton, Marcella Ferrero, Neil Flack, Howerd Fordham, Grace Forsythe, Matt Francis, Audrey Fraser, Adam Freeman, Anastasia Galvin, Maria Garcia-Casado, Alex Gedny, Sophia Girgis, James Glover, Oliver Gould, Andy Gray, Emma Gray, Coline Griffiths, Yong Gu, Florence Guerin, Will Hamilton, Hannah Hanks, Ewan Harrison, Alexandria Harrott, Edward Harry, Julia Harvison, Paul Heath, Anastasia Hernandez-Koutoucheva, Rhiannon Hobbs, Dave Holland, Sarah Holmes, Gary Hornett, Nicholas Hough, Liz Huckle, Lena Hughes-Hallet, Adam Hunter, Stephen Inglis, Sameena Iqbal, Adam Jackson, David Jackson, Carlos Jimenez Verdejo, Matthew Jones, Kalyan Kallepally, Keely Kay, Jon Keatley, Alan Keith, Alison King, Lucy Kitchin, Matt Kleanthous, Martina Klimekova, Petra Korlevic, Ksenia Krasheninnkova, Greg Lane, Cordelia Langford, Adam Laverack, Katharine Law, Stefanie Lensing, Amanah Lewis-Wade, Jennifer Liddle, Quan Lin, Sarah Lindsay, Sally Linsdell, Rhona Long, Jamie Lovell, Jon Lovell, James Mack, Mark Maddison, Aleksei Makunin, Irfan Mamun, Jenny Mansfield, Neil Marriott, Matt Martin, Matthew Mayho, Jo McClintock, Sandra McHugh, Liz McMinn, Carl Meadows, Emily Mobley, Robin Moll, Maria Morra, Leanne Morrow, Kathryn Murie, Sian Nash, Claire Nathwani, Plamena Naydenova, Alexandra Neaverson, Ed Nerou, Jon Nicholson, Tabea Nimz, Guillaume G. Noell, Sarah O’Meara, Valeriu Ohan, Charles Olney, Doug Ormond, Agnes Oszlanczi, Yoke Fei Pang, Barbora Pardubska, Naomi Park, Aaron Parmar, Gaurang Patel, Maggie Payne, Sharon Peacock, Arabella Petersen, Deborah Plowman, Tom Preston, Michael Quail, Richard Rance, Suzannah Rawlings, Nicholas Redshaw, Joe Reynolds, Mark Reynolds, Simon Rice, Matt Richardson, Connor Roberts, Katrina Robinson, Melanie Robinson, David Robinson, Hazel Rogers, Eduardo Martin Rojo, Daljit Roopra, Mark Rose, Luke Rudd, Ramin Sadri, Nicholas Salmon, David Saul, Frank Schwach, Phil Seekings, Alison Simms, Matt Sinnott, Shanthi Sivadasan, Bart Siwek, Dale Sizer, Kenneth Skeldon, Jason Skelton, Joanna Slater-Tunstill, Lisa Sloper, Nathalie Smerdon, Chris Smith, Christen Smith, James Smith, Katie Smith, Michelle Smith, Sean Smith, Tina Smith, Leighton Sneade, Carmen Diaz Soria, Catarina Sousa, Emily Souster, Andrew Sparkes, Michael Spencer-Chapman, Janet Squares, Robert Stanley, Claire Steed, Tim Stickland, Ian Still, Mike Stratton, Michelle Strickland, Allen Swann, Agnieszka Swiatkowska, Neil Sycamore, Emma Swift, Edward Symons, Suzanne Szluha, Emma Taluy, Nunu Tao, Katy Taylor, Sam Taylor, Stacey Thompson, Mark Thompson, Mark Thomson, Nicholas Thomson, Scott Thurston, Dee Toombs, Benjamin Topping, Jaime Tovar-Corona, Daniel Ungureanu, James Uphill, Jana Urbanova, Philip Jansen Van, Valerie Vancollie, Paul Voak, Danielle Walker, Matthew Walker, Matt Waller, Gary Ward, Charlie Weatherhogg, Niki Webb, Alan Wells, Eloise Wells, Luke Westwood, Theo Whipp, Thomas Whiteley, Georgia Whitton, Sara Widaa, Mia Williams, Mark Wilson, and Sean Wright.

## STAR★Methods

### Key resources table

**Table:** 

REAGENT or RESOURCE	SOURCE	IDENTIFIER
**Antibodies**		

Anti-HIV p24	Abcam	Cat#Ab9071
Anti-FLAG	Sigma Aldrich	Cat#F7425
Anti-SARS-CoV-2 spike	Novusbio	Cat#NB100-56578
RNA (MS2)	Roche	Cat#10165948001
HIV RT	Milipore	Cat#382129
Anti-rabbit HRP conjugate	Cell Signaling	Cat#7074

**Bacterial and virus strains**		

XL1-blue cells	Agilent	Cat#200249

**Chemicals, peptides, and recombinant proteins**		

*Trans*IT-X2	Mirus	Cat#MIR 6000
Polyethylenimine (PEI)	Sigma Aldrich	Cat#408727

**Critical commercial assays**		

Bright-Glo	Promega	Cat#E2650
QuikChange Lightning	Agilent	Cat#210518
Luna Universal qPCR Master Mix	New England Biolabs	Cat#M3003L

**Experimental models: Cell lines**		

HEK293T	ATCC	Cat#CRL-3216

**Oligonucleotides**		

MS2 FP: 5′-TCCTGCTCAACTTCCTGTCGAG-3′	This paper	N/A
MS2 RP: 5′-CACAGGTCAAACCTCCTAGGAATG-3′	This paper	N/A
Spike_Δ69/70 FP1: 5′- GGTTCCACGCCATCAGCGGCACAAACGG-3′	This paper	N/A
Spike_Δ69/70 FP2: 5′- GCGCTAATTTAAGCTTGCCACCATGTTCGTG −3′	This paper	N/A
Spike_Δ69/70 RP1: 5′- CCGTTTGTGCCGCTGATGGCGTGGAACC −3′	This paper	N/A
Spike_Δ69/70 RP2: 5′- TAATGGGTCCCTCACGGCGTCGGTTG −3′	This paper	N/A
Spike_furin_KO_FP1: 5′-CTGGCTAGCGTTTAAACTTAGCCACCATGTTCGTGTTC-3′	This paper	N/A
Spike_furin_KO_FP2: 5′- *GGCCCGCCGAGG*GGAGTTTGTCTGGGTCTG-3′	This paper	N/A
Spike_furin_KO_RP1: 5′-GACAAACTCC*CCTCGGCGGGCC* CGGAGCGTGGCCAGCCAG −3′	This paper	N/A
Spike_furin_KO_RP2: 5′-CGGGCCCTCTAGACTCGAGC GCCTACTTATCATCATCATCCTTATAGTCAGTGTAGTGC-3′	This paper	N/A

**Recombinant DNA**		

Plasmid: SARS-CoV-2 spike D614-FLAG	Biobasic	N/A
Plasmid: RaTG13 spike-FLAG	Biobasic	N/A
Plasmid: human ACE2 receptor	Biobasic	N/A
Plasmid: TMPRSS2	Biobasic	N/A
Plasmid: p8.91	This paper	N/A
Plasmid: CSFLW	This paper	N/A
Plasmid: pcDNA3.1	Thermo Scientific, Invitrogen	Cat#V66020

**Software and algorithms**		

Pangolin v2.4.2	Rambaut et al., 2020a	https://github.com/cov-lineages/pangolin
IQTREE2 v2.1.2	Minh et al., 2020	http://www.iqtree.org/
ModelFinder	Kalyaanamoorthy et al., 2017	NA
Figtree v1.4.4	Rambaut, 2012	http://tree.bio.ed.ac.uk/software/figtree/
RDP5	Martin et al., 2020	http://web.cbio.uct.ac.za/∼darren/rdp.html
RAXML-NG v1.02	Kozlov et al., 2019	https://github.com/amkozlov/raxml-ng
BioEdit v7.2	Bhullar et al., 2012	NA
RNAalifold	Bernhart et al., 2008	http://rna.tbi.univie.ac.at/cgi-bin/RNAWebSuite/RNAalifold.cgi
I-TASSER	Roy et al., 2010	
Pymol v2.4.0	Schrödinger Inc., New York, USA	https://github.com/schrodinger/pymol-open-source

**Other**		

Sequence data from the GISAID public database	(Shu and McCauley, 2017)	https://gisaid.org/

### Resource availability

#### Lead contact

Further information should be directed to and will be fulfilled by the Lead Contact, Ravindra K. Gupta rkg20@cam.ac.uk.

#### Materials availability

This study did not generate new unique reagents.

#### Data and code availability

Raw anonymized data are available from the lead contact without restriction.

### Experimental model and subject details

The study was primarily a laboratory based study using pseudotyped virus (PV) with mutations generates by site directed mutagenesis. We tested infectivity in cell lines with a range of drug inhibitors and monoclonal antibodies. Sensitivity to antibodies in serum was tested using convalescent sera from recovered individuals collected as part of the Cambridge NIHR Bioresource. We also performed phylogenetic analyses of data available publicly in GISAID.

#### Ethical approval

Ethical approval for use of serum samples. Controls with COVID-19 were enrolled to the NIHR BioResource Centre Cambridge under ethics review board (17/EE/0025).

### Method details

#### Phylogenetic analysis

All available full-genome SARS-CoV-2 sequences were downloaded from the GISAID database (https://gisaid.org/; Shu and McCauley, 2017) on 16^th^ February 2021. Low-quality sequences (> 5% N regions) were removed, leaving a dataset of 491,395 sequences with a length of > 29,000bp. Sequences were deduplicated and then filtered to find the mutations of interest. All sequences were realigned to the SARS-CoV-2 reference strain MN908947.3, using MAFFT v7.475 with automatic strategy selection and the–keeplength–addfragments options (Katoh and Standley, 2013). Major SARS-CoV-2 clade memberships were assigned to all sequences using the Nextclade server v0.13 (https://clades.nextstrain.org/), Pangolin v2.4.2 (Rambaut et al., 2020a); https://github.com/cov-lineages/pangolin) and a local instance of the PangoLEARN model, dated 18^th^ April 21:49 (https://github.com/cov-lineages/pangoLEARN).

Maximum likelihood phylogenetic trees were produced using the above curated dataset using IQ-TREE v2.1.2 (Minh et al., 2020). Evolutionary model selection for trees were inferred using ModelFinder (Kalyaanamoorthy et al., 2017) and trees were estimated using the GTR+F+I model with 1000 ultrafast bootstrap replicates (Minh et al., 2013). All trees were visualized with Figtree v.1.4.4 (http://tree.bio.ed.ac.uk/software/figtree/) and ggtree v2.2.4 rooted on the SARS-CoV-2 reference sequence and nodes arranged in descending order. Nodes with bootstraps values of < 50 were collapsed using an in-house script.

To reconstruct a phylogeny for the 69/70 spike region of the 20 *Sarbecoviruses* examined in Figure S4, Rdp5 (Martin et al., 2015) was used on the codon spike alignment to determine the region between amino acids 1 and 256 as putatively non-recombinant. A tree was reconstructed using the nucleotide alignment of this region under a GTR+Γ substitution model with RAxML-NG (Kozlov et al., 2019). Node support was calculated with 1000 bootstraps. Alignment visualization was done using BioEdit (Hall et al., 2011).

#### Structural modeling

The structure of the post-deletion NTD (residues 14-306) was modeled using I-TASSER (Roy et al., 2010), a method involving detection of templates from the protein data bank, fragment structure assembly using replica-exchange Monte Carlo simulation and atomic-level refinement of structure using a fragment-guided molecular dynamics simulation. The structural model generated was aligned with the spike structure possessing the pre-deletion conformation of the 69-77 loop (PDB 7C2L (Chi et al., 2020)) using PyMOL (Schrödinger). Figures prepared with PyMOL using PDBs 7C2L, 6M0J (Lan et al., 2020), 6ZGE28 and 6ZGG (Wrobel et al., 2020).

#### RNA secondary structure modeling

2990 nucleotides centered around the spike protein amino acids 69-70 from SARS-CoV2 sequence from an individual^12^ were aligned in CLUSATL-Omega (nucleotides 20277-23265 of the Wuhan isolate MN908947.3) and a consensus structure was generated using RNAalifold (Bernhart et al., 2008)).

#### Cells

HEK293T CRL-3216, Vero CCL-81 were purchased from ATCC and maintained in Dulbecco’s Modified Eagle Medium (DMEM) supplemented with 10% fetal calf serum (FCS), 100 U/ml penicillin, and 100mg/ml streptomycin. All cells are regularly tested and are mycoplasma free. H1299 cells were a kind gift from Simon Cook. Calu-3 cells were a kind gift from Paul Lehner, A549 A2T2 (Rihn et al., 2021) cells were a kind gift from Massimo Palmerini.

#### Pseudotype virus preparation

Plasmids encoding the spike protein of SARS-CoV-2 D614 with a C-terminal 19 amino acid deletion with D614G, were used as a template to produce variants lacking amino acids at position H69 and V70, as well as mutations N439K and Y453F. Mutations were introduced using Quickchange Lightning Site-Directed Mutagenesis kit (Agilent) following the manufacturer’s instructions. B.1.1.7 S expressing plasmid preparation was described previously by step wise mutagenesis (Collier et al., 2021). Viral vectors were prepared by transfection of 293T cells by using Fugene HD transfection reagent (Promega). 293T cells were transfected with a mixture of 11ul of Fugene HD, 1μg of pCDNAΔ19 spike-HA, 1ug of p8.91 HIV-1 gag-pol expression vector and 1.5μg of pCSFLW (expressing the firefly luciferase reporter gene with the HIV-1 packaging signal). Viral supernatant was collected at 48 and 72h after transfection, filtered through 0.45um filter and stored at −80°C as previously described. Infectivity was measured by luciferase detection in target 293T cells transfected with TMPRSS2 and ACE2.

#### SARS-CoV-2 D614 (Wuhan) and RaTG13 mutant plasmids and infectivity

Plasmids encoding the full-length spike protein of SARS-CoV-2 D614 (Wuhan) and RaTG13, in frame with a C – terminal Flag tag (Conceicao et al., 2020), were used as a template to produce variants lacking amino acids at position H69 and V70. The deletion was introduced using Quickchange Lightning Site-Directed Mutagenesis kit (Agilent) following the manufacturer’s instructions. Viruses were purified by ultracentrifugation; 25mL of crude preparation being purified on a 20% sucrose cushion at 2300rpm for 2 hr at 4°C. After centrifugation, the supernatant was discarded and the viral pellet resuspended in 600 μL DMEM (10% FBS) and stored at −80°C. Infectivity was examined in HEK293 cells transfected with human ACE2, with RLUs normalized to RT activity present in the pseudotyped virus preparation by PERT assay. Western blots were performed on purified virus with anti-HIV1 p24, 1:1,000 (Abcam) or anti-FLAG, 1:2,000 (Sigma) antibodies used following SDS-PAGE and transfer.

#### Standardization of virus input by SYBR green-based product-enhanced PCR assay (SG-PERT)

The reverse transcriptase activity of virus preparations was determined by qPCR using a SYBR Green-based product-enhanced PCR assay (SG-PERT) as previously described (Vermeire et al., 2012). Briefly, 10-fold dilutions of virus supernatant were lysed in a 1:1 ratio in a 2x lysis solution (made up of 40% glycerol v/v 0.25% Trition X-100 v/v 100mM KCl, RNase inhibitor 0.8 U/ml, TrisHCL 100mM, buffered to pH7.4) for 10 minutes at room temperature.

12μl of each sample lysate was added to 13μl of a SYBR Green master mix (containing 0.5μM of MS2-RNA Fwd and Rev primers, 3.5pmol/ml of MS2-RNA, and 0.125U/μl of Ribolock RNase inhibitor and cycled in a QuantStudio. Relative amounts of reverse transcriptase activity were determined as the rate of transcription of bacteriophage MS2 RNA, with absolute RT activity calculated by comparing the relative amounts of RT to an RT standard of known activity.

#### Generation of coronavirus-like particles and western blotting

Plasmids encoding codon-optimized (M)embrane, (E)nvelope and (N)ucleocapsid proteins of SARS-CoV-2 were a kind gift from Nevan Krogan (M, pLVX-EF1alpha-SARS-CoV-2-M-2xStrep-IRES-Puro, Addgene #141386; E, pLVX-EF1alpha-SARS-CoV-2-E-2xStrep-IRES-Puro, Addgene #141385; N, pLVX-EF1alpha-SARS-CoV-2-N-2xStrep-IRES-Puro, Addgene #141391) (Gordon et al., 2020). For expression in 293T cells, M, E and N were each subcloned into a modified pmaxGFP (Lonza) vector as M/E/N-IRES-GFP. Plasmids encoding the (S)pike protein of SARS-CoV-2 with a C-terminal 19 amino acid deletion plus/minus the ΔH69/V70 deletion are described above (pseudotype virus preparation).

Coronavirus-like particles were prepared essentially as previously described (Swann et al., 2020; Yurkovetskiy et al., 2020). In brief, 4 ×10^6^ 293T cells in 10 cm dishes were transfected using TransIT-LT1 (Mirus) with a total of 4 μg DNA comprising 1 μg each of plasmids encoding S (WT or ΔH69/V70), M, E and N. Media was replaced after 16 h.

Supernatants containing coronavirus-like particles were harvested after 2 d, spun for 10 min at 2,000 g, then passed through a 0.45 μm filter. For each condition, 9 mL supernatant was layered on a 2 mL cushion of 20% sucrose in PBS and spun for 2 h at 100,000 g in a Type 70 Ti Beckman Coulter Ultracentrifuge Rotor. The pellet was washed once with PBS, then resuspended in 100 μl 2% SDS in PBS. After aspiration of supernatant, cells were washed twice in PBS, then lysed in 800 μl of 2% SDS in TBS with 500 units of Benzonase (Sigma-Aldrich). Lysates were incubated for 30 m at room temperature, then spun for 10 min at 13,000 g.

Resuspended pellets containing concentrated coronavirus-like particles were heated in Laemmli buffer with DTT at 95°C for 5 min. For each condition, 30 μL was loaded on a 4%–20% Mini-PROTEAN TGX Precast Protein Gel (Bio-Rad). Cell lysates were quantified using the Pierce BCA Protein Assay Kit (Thermo Scientific), then heated in Laemmli buffer with DTT at 95°C for 5 min. For each condition, 20 μg protein was loaded on an identical gel. Proteins were transferred to 45 nm PDVF membranes and blocked with 5% milk in PBS-Tween 0.2%. The following antibodies were used for immunoblotting: anti-S (Invitrogen, PA1-41165); anti-N (Novus Biologicals, NB100-56683) and anti-β-actin (Sigma, A5316).

#### Spike cleavage inhibition experiments

CMK furin inhibitor experiments: 293T cells were transfected with plasmids expressing Gag/pol, luciferase, and spike. Furin inhibitor CMK (Calbiochem) was added at either 5 M or 25 μM concentration three hours post transfection. The supernatants and cell lysates were collected after 48 hours for infectivity on target cells or for western blotting.

E64D and camostat experiments: ACE2 or ACE2 and TMPRSS2 transfected 293T cells were either E64D (Tocris) or camostat (Sigma-Aldrich) treated for 3 hours at each drug concentration before the addition of a comparable amount of input viruses pseudotyped with WT, H69/V70 deletion or VSV-G (approx. 1 million RLU). The cells were then left for 48 hours before addition of substrate for luciferase (Promega) and read on a Glomax plate reader (Promega). The RLU was normalized against the no-drug control which was set as 100%.

#### Cell-cell fusion assay

Cell fusion assay was carried out as previously described (Papa et al., 2021). Briefly, Vero cells and 293T cells were seeded at 80% confluency in a 24 multiwell plate. 293T cells were co-transfected with 1.5 μg of spike expression plasmids in pCDNA3 and 0.5 μg mCherry-N1 using Fugene 6 and following the manufacturer’s instructions (Promega). Vero cells were treated with CellTracker Green CMFDA (5-chloromethylfluorescein diacetate) (Thermo Scientific) for 20 minutes. 293T cells were then detached 5 hours post transfection, mixed together with the green-labeled Vero cells, and plated in a 12 multiwell plate. Cell-cell fusion was measured using an Incucyte and determined as the proportion of merged area to green area over time. Data were then analyzed using Incucyte software analysis. Data were normalized to cells transfected only with mCherry protein and mixed with green labeled Vero cells. Graphs were generated using Prism 8 software.

#### Western blotting

Cells were lysed and supernatants collected 18 hours post transfection. Purified virions were prepared by harvesting supernatants and passing through a 0.45 μm filter. Clarified supernatants were then loaded onto a thin layer of 8.4% optiprep density gradient medium (Sigma-Aldrich) and placed in a TLA55 rotor (Beckman Coulter) for ultracentrifugation for 2 hours at 20,000 rpm. The pellet was then resuspended for western blotting. Cells were lysed with cell lysis buffer (Cell signaling) or were treated with Benzonase Nuclease (Millipore) and boiled for 5 min. Samples were then run on 4%–12% Bis Tris gels and transferred onto nitrocellulose or PVDF membranes using an iBlot or semidry (Thermofisher and Biorad, respectively).

Membranes were blocked for 1 hour in 5% non-fat milk in PBS + 0.1% Tween-20 (PBST) at room temperature with agitation, incubated in primary antibody (anti-SARS-CoV-2 Spike, Thermofisher, PA1-41165), anti-GAPDH (proteintech) or anti-p24 (NIBSC)) diluted in 5% non-fat milk in PBST for 2 hours at 4°C with agitation, washed four times in PBST for 5 minutes at room temperature with agitation and incubated in secondary antibody (anti-rabbit or anti-mouse HRP conjugate), anti-bactin HRP (Santa Cruz) diluted in 5% non-fat milk in PBST for 1 hour with agitation at room temperature. Membranes were washed four times in PBST for 5 minutes at room temperature and imaged directly using a ChemiDoc MP imaging system (Bio-Rad).

#### Serum pseudotype neutralization assay

Spike pseudotype assays have been shown to have similar characteristics as neutralization testing using fully infectious wild-type SARS-CoV-2 (Schmidt et al., 2020).Virus neutralization assays were performed on 293T cell transiently transfected with ACE2 and TMPRSS2 using SARS-CoV-2 spike pseudotyped virus expressing luciferase (Mlcochova et al., 2020). Pseudotyped virus was incubated with serial dilution of heat inactivated human serum samples or convalescent plasma in duplicate for 1h at 37°C. Virus and cell only controls were also included. Then, freshly trypsinized 293T ACE2/TMPRSS2 expressing cells were added to each well. Following 48h incubation in a 5% CO_2_ environment at 37°C, the luminescence was measured using Steady-Glo Luciferase assay system (Promega).

#### Monoclonal antibody neutralization of B.1.1.7 or B.1.1.7 H69/V70 pseudotyped viruses

Preparation of B.1.1.7 or B.1.1.7 H69/V70 SARS-CoV-2 S glycoprotein-encoding-plasmid used to produce SARS-CoV-2-MLV based on overlap extension PCR. Briefly, a modification of the overlap extension PCR protocol (Forloni et al., 2018) was used to introduce the 9 or 7 mutations of the B.1.1.7 and B.1.1.7 H69/V70 lineages, respectively. In a first step, 9 DNA fragments with overlap sequences were amplified by PCR from a plasmid (phCMV1, Genlantis) encoding the full-length SARS-CoV-2 S gene (BetaCoV/Wuhan-Hu-1/2019, accession number mn908947). The mutations (Δ69/70, Δ144, N501Y, A570D, D614G, P681H, S982A, T716I and D1118H or K417N, E484K and N501Y) were introduced by amplification with primers with similar Tm. Deletion of the C-terminal 21 amino acids was introduced to increase surface expression of the recombinant S (Case et al., 2020). Next, 3 contiguous overlapping fragments were fused by a first overlap PCR (step 2) using the utmost external primers of each set, resulting in 3 larger fragments with overlapping sequences. A final overlap PCR (step 3) was performed on the 3 large fragments using the utmost external primers to amplify the full-length S gene and the flanking sequences including the restriction sites KpnI and NotI. This fragment was digested and cloned into the expression plasmid phCMV1. For all PCR reactions the Q5 Hot Start High fidelity DNA polymerase was used (New England Biolabs Inc.), according to the manufacturer’s instructions and adapting the elongation time to the size of the amplicon. After each PCR step the amplified regions were separated on agarose gel and purified using Illustra GFX PCR DNA and Gel Band Purification Kit (Merck KGaA).

#### Ab discovery and recombinant expression

Human mAbs were isolated from plasma cells or memory B cells of SARS-CoV or SARS-CoV-2 immune donors. Recombinant antibodies were expressed in ExpiCHO cells at 37°C and 8% CO2. Cells were transfected using ExpiFectamine. Transfected cells were supplemented 1 day after transfection with ExpiCHO Feed and ExpiFectamine CHO Enhancer. Cell culture supernatant was collected eight days after transfection and filtered through a 0.2 μm filter. Recombinant antibodies were affinity purified on an ÄKTA xpress FPLC device using 5 mL HiTrap MabSelect PrismA columns followed by buffer exchange to Histidine buffer (20 mM Histidine, 8% sucrose, pH 6) using HiPrep 26/10 desalting columns.

#### Pseudovirus neutralization assay for testing NTD monoclonal antibodies

MLV-based SARS-CoV-2 S-glycoprotein-pseudotyped viruses were prepared as previously described (Pinto, 2020). HEK293T/17cells were cotransfected with a WT, B.1.1.7 or B.1.1.7 H69/V70 SARS-CoV-2 spike glycoprotein-encoding-plasmid, an MLV Gag-Pol packaging construct and the MLV transfer vector encoding a luciferase reporter using X-tremeGENE HP transfection reagent (Roche) according to the manufacturer’s instructions. Cells were cultured for 72 h at 37°C with 5% CO_2_ before harvesting the supernatant. VeroE6 stably expressing human TMPRSS2 were cultured in Dulbecco’s Modified Eagle’s Medium (DMEM) containing 10% fetal bovine serum (FBS), 1% penicillin–streptomycin (100 I.U. penicillin/mL, 100 μg/mL), 8 μg/mL puromycin and plated into 96-well plates for 16–24 h. Pseudovirus with serial dilution of mAbs was incubated for 1 h at 37°C and then added to the wells after washing 2 times with DMEM. After 2–3 h DMEM containing 20% FBS and 2% penicillin–streptomycin was added to the cells. Following 48-72 h of infection, Bio-Glo (Promega) was added to the cells and incubated in the dark for 15 min before reading luminescence with Synergy H1 microplate reader (BioTek). Measurements were done in duplicate and relative luciferase units were converted to percent neutralization and plotted with a non-linear regression model to determine IC_50_ values using GraphPad PRISM software (version 9.0.0).

#### Neutralization assays

Measurements were done in duplicate and relative luciferase units were converted to percent neutralization against no-drug control which was set as 100%. Data were plotted with a non-linear regression model to determine IC_50_ values using GraphPad PRISM software (version 9.0.0). The 50% inhibitory dilution (EC_50_) was defined as the serum dilution at which the relative light units (RLUs) were reduced by 50% compared with the virus control wells (virus + cells) after subtraction of the background RLUs in the control groups with cells only. The EC_50_ values were calculated with non-linear regression, log (inhibitor) versus normalized response using GraphPad Prism 8 (GraphPad Software, Inc., San Diego, CA, USA). The neutralization assay was positive if the serum achieved at least 50% inhibition at 1 in 3 dilution of the SARS-CoV-2 spike protein pseudotyped virus in the neutralization assay. The neutralization result was negative if it failed to achieve 50% inhibition at 1 in 3 dilution. Statistical tests are described in the figure legends along with the value of n, mean, and standard deviation/error. Data were normally distributed consistent with statistical methods used.

### Quantification and statistical analysis

Measurements were done in duplicate and relative luciferase units measured with a Glomax luminometer. Data were analyzed using GraphPad PRISM software (version 9.0.0). Statistical tests are described in the figure legends along n, mean, and standard deviation/error. Data were normally distributed consistent with statistical methods used.

## Supplemental information

Document S1. Figures S1–S5

Document S2. Article plus supplemental information
