# Supplementary material for: Recurrent emergence of SARS-CoV-2 spike deletion H69/V70 and its role in the Alpha variant B.1.1.7
Source: Cell Rep. 2021 Jun 8;35(13):109292. doi: 10.1016/j.celrep.2021.109292 (PMC8185188; doi:10.1016/j.celrep.2021.109292)
Supplement: Document S1. Figures S1–S5 [file mmc1.pdf]

Cell Reports, Volume *35*

Supplemental information

Recurrent emergence of SARS-CoV-2 spike deletion H69/V70 and its role in the Alpha variant B.1.1.7

Bo Meng, Steven A. Kemp, Guido Papa, Rawlings Datir, Isabella A.T.M. Ferreira, Sara Marelli, William T. Harvey, Spyros Lytras, Ahmed Mohamed, Giulia Gallo, Nazia Thakur, Dami A. Collier, Petra Mlcochova, The COVID-19 Genomics UK (COG-UK) Consortium, Lidia M. Duncan, Alessandro M. Carabelli, Julia C. Kenyon, Andrew M. Lever, Anna De Marco, Christian Saliba, Katja Culap, Elisabetta Cameroni, Nicholas J. Matheson, Luca Piccoli, Davide Corti, Leo C. James, David L. Robertson, Dalan Bailey, and Ravindra K. Gupta

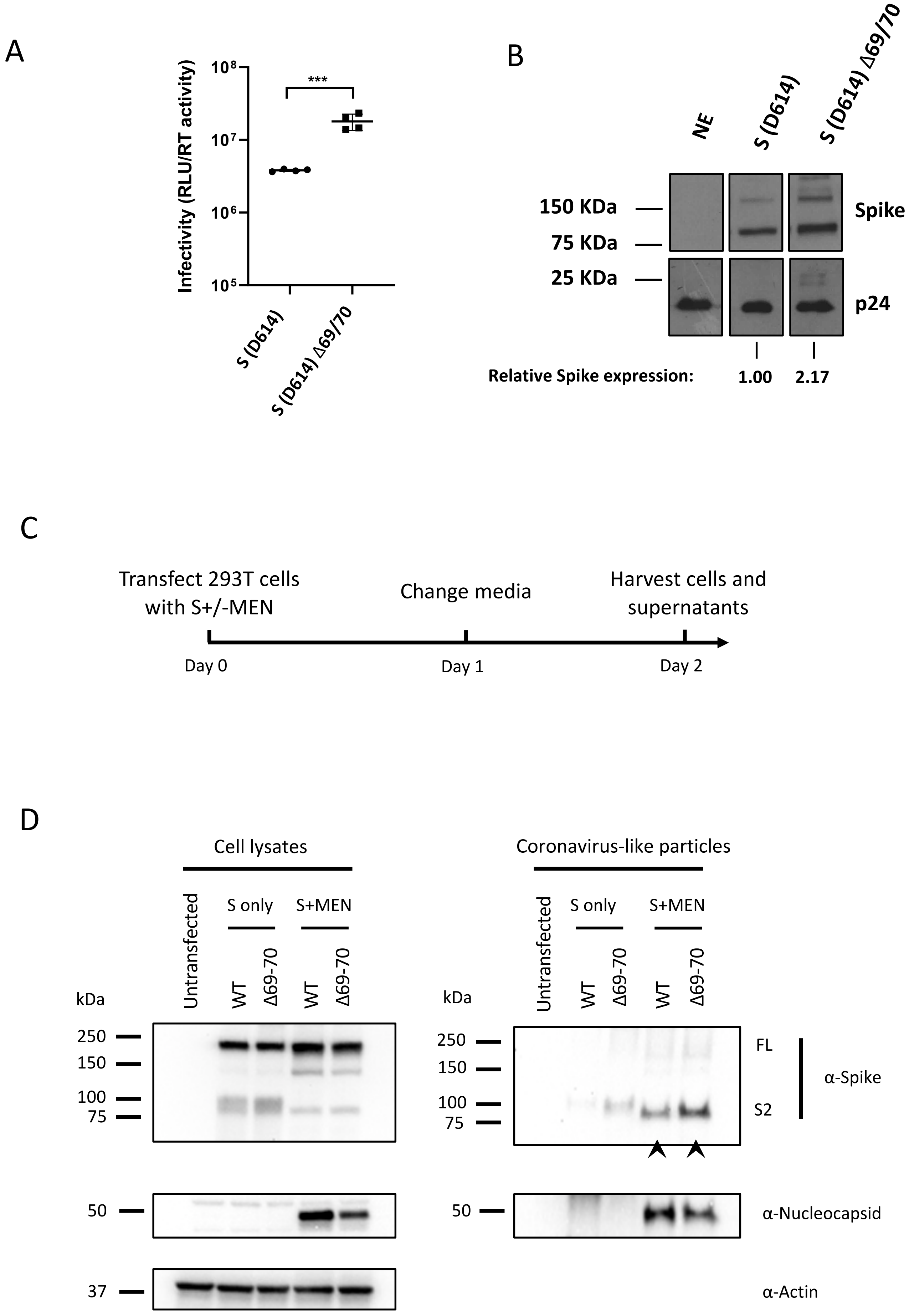

Supplementary Figure 1: **Infectivity and cleavage of spike ΔH69/V70 in a background of D614 (Wuhan) in pseudotyped lentivirus.** Sucrose purified pseudotypes, as indicated, were used to infect human ACE2 expressing HEK293 cells, with luciferase readings read at 72 hours post infection. Experiments were performed in biological quadruplicate with the mean and standard deviation plotted. Results are representative of experiments performed two times. Statistical significance was assessed using an unpaired t-test (ns; non-significant, ***; <0.005). Western blot of purified pseudotype virus. Spike and HIV pseudotype abundances were assessed using Flag and p24 antibodies, respectively. Relative spike expression was calculated by densitometry using Image J. Briefly, inverted pixel intensities for spike and p24 bands were first normalised to a background region of the gel. Spike protein intensities were then normalised to p24 intensity before mutant protein expression was calculated as a factor of wild-type protein. NE: no envelope/spike. **C, D. Cleavage of spike ΔH69/V70 versus WT (D614G background) in coronavirus like particles. C.** Generation of coronavirus-like particles. Supernatants containing coronavirus-like particles were concentrated by ultracentrifugation, then analysed by immunoblot in parallel with lysates from producer cells **D**. Immunoblots of cell lysates and coronavirus-like particles from (A). 293T cells were transfected with plasmids encoding the indicated SARS-CoV-2 proteins (S, spike, WT or ΔH69/V70; M, membrane; E, envelope; N, nucleocapsid). Cells transfected with WT or ΔH69/V70 S alone (no M, E or N) were included as controls. Arrow heads indicate cleaved S in coronavirus-like particles. Representative data are shown from three independent experiments. Related to Figure 3.

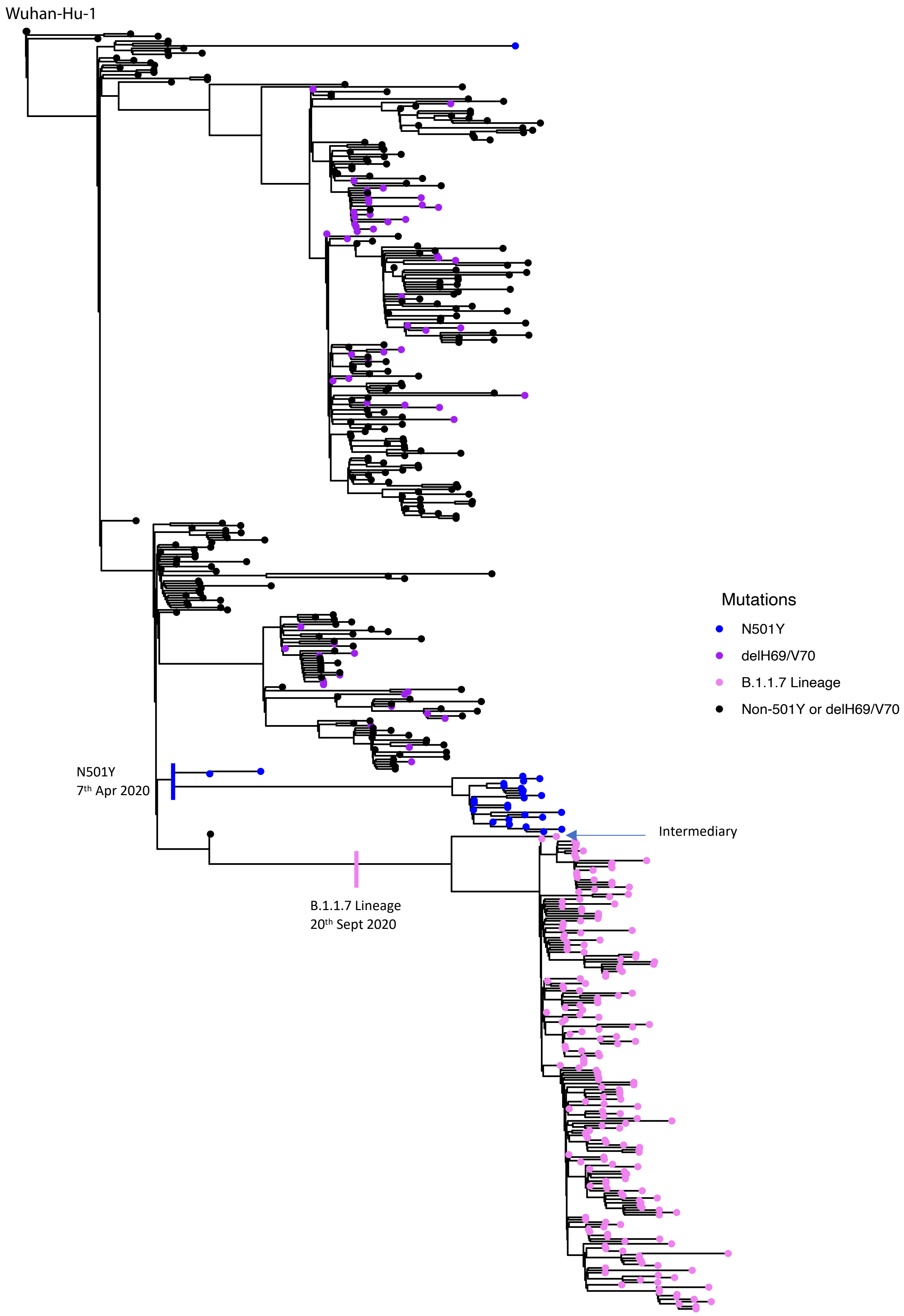

Supplementary Figure 2: Maximum likelihood phylogeny of UK viruses bearing Δ69/70 and N501Y mutations. Two distinct lineages of the ΔH69/V70 were observed to expand in the UK, separately from the 501Y lineage. Prior to expansion of the B.1.1.7 lineage, clusters of infections bearing either N501Y or ΔH69/V70 were observed. Alongside expansion of the B.1.1.7 lineage, is a population in Wales that carries 501Y, but no ΔH69/V70. An intermediary was detected alongside the B.1.1.7 lineage (indicated on phylogeny) which had only a subset of the mutations that make up B.1.1.7 (ΔH69/V70, N501Y, A570D and D1118H). Related to Figure 6.

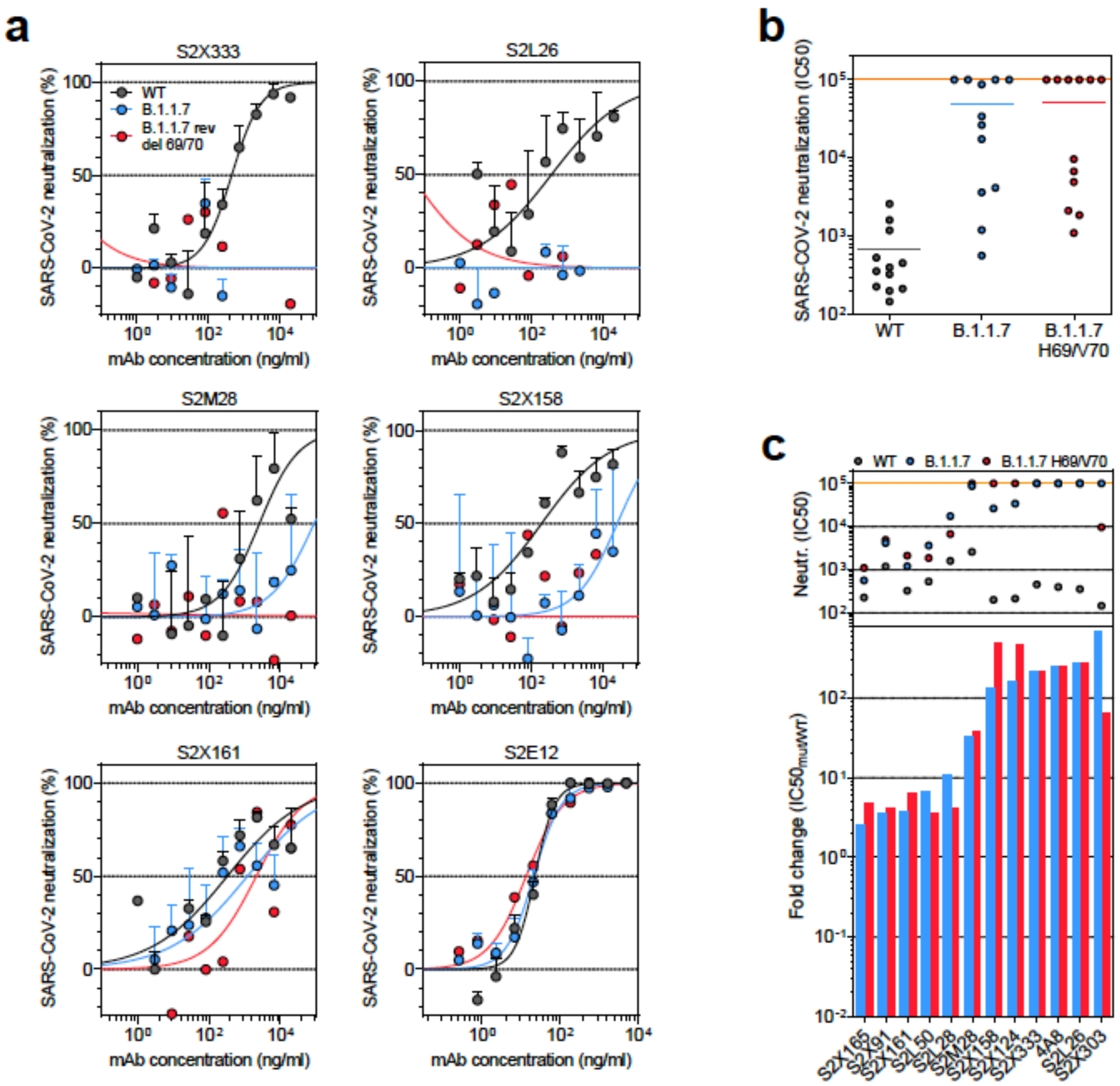

Supplementary Figure 3: Neutralisation and binding by a panel of NTD-specific mAbs against WT, B.1.1.7 and B.1.1.7 H69/V70 mutant SARS-CoV-2 viruses. A. Neutralisation of WT (black), B.1.1.7 (blue) and B.1.1.7 H69/V70 replacement mutant (red) pseudotyped SARS-CoV-2-MLVs by 6 selected mAbs from one experiment. **B.** Neutralisation of WT, B.1.1.7 and B.1.1.7 H69/V70 SARS-CoV-2-MLVs by 13 mAbs targeting NTD. Shown are the mean IC50 values (ng/ml) from one experiment. The higher the IC50 the less sensitive the virus to antibodies. **C.** Neutralisation shown as mean IC50 values (upper panel) and mean fold change of B.1.1.7 (blue) or B.1.1.7 H69/V70 (red) relative to WT (lower panel) of the 13 NTD mAbs tested. Lower panel shows IC50 values from one experiment. Related to Figure 6.

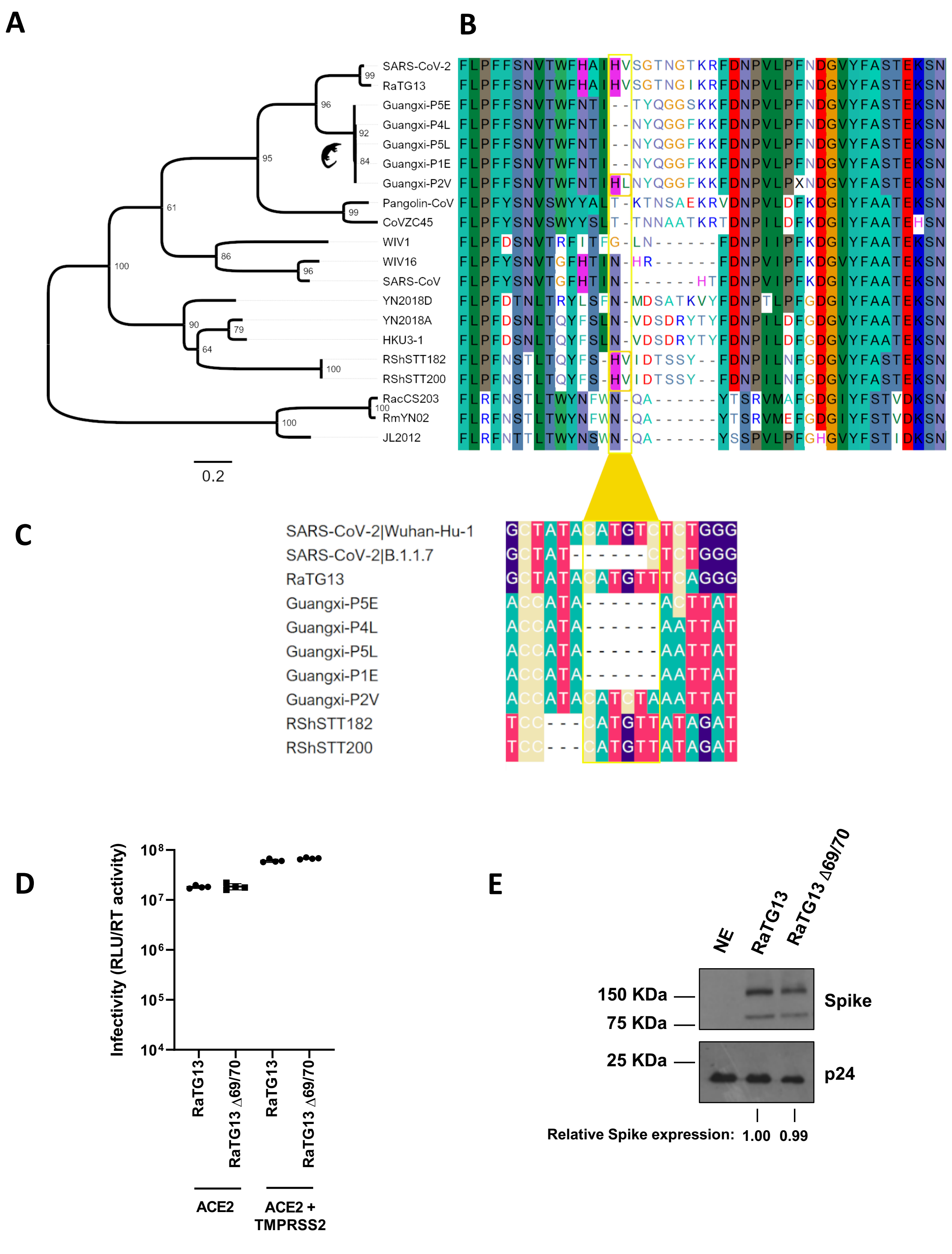

Supplementary Figure 4. Comparison of the H69/V70 deletion site to other *Sarbecoviruses*. A. phylogeny for the Spike peptide region 1-256 **B**. protein sequences from 20 Sarbecoviruses, including SARS-CoV-2 (Wuhan-Hu-1) and SARS-CoV (HSZ-Cc), with distinct genotypes at the Spike region around amino acid positions 69 and 70 (highlighted in yellow box). The 69/70 HL insertion in the P2V sequence from the Guangxi pangolin virus cluster and the HV convergent insertion in the RShSTT182/200 bat virus sequences are highlighted. **C**. The nucleotide alignment between SARS-CoV-2 Wuhan-Hu-1, B.1.1.7, the bat sarbecovirus RaTG13 RShSTT182/200 and the Guangxi pangolin viruses shows the difference between the out-of-frame deletion observed in the former and the in-frame deletion in the latter. **D.** Single round infection by luciferase expressing lentivirus pseudotyped with RaTG13 Spike protein on 293T cells transduced with ACE2**.** Experiments were performed in biological quadruplicate with the mean and standard deviation plotted. Results are representative of experiments performed two times. Statistical significance was assessed using an unpaired t-test (ns; non-significant, ***; <0.005). **E**. Representative western blot of supernatant from virus producer cells. Spike and HIV pseudotype abundances were assessed using Flag and p24 antibodies, respectively. Relative spike expression (total spike:p24) was calculated by densitometry using Image J. NE: no envelope. Relates to Figure 7.

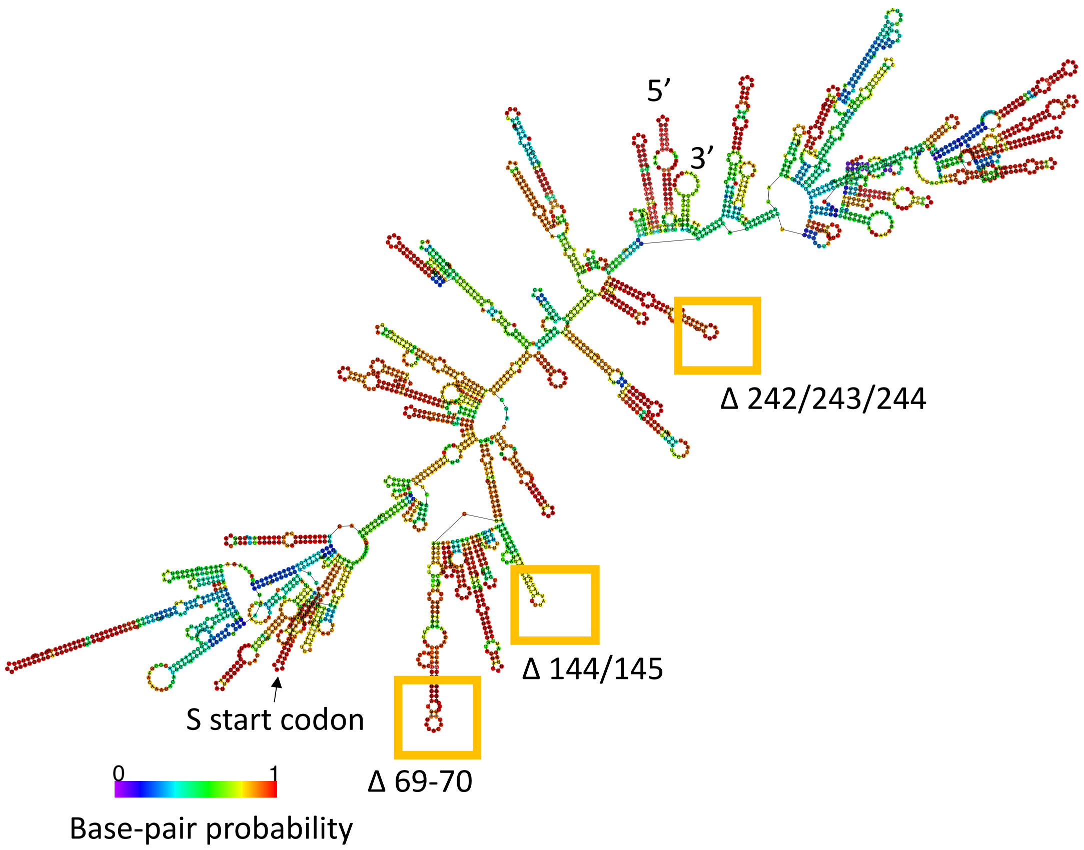

Supplementary Figure 5: The positions of common deletion mutations on the RNA structure of the Spike Δ69-70 region of the gRNA. The optimal secondary structure was generated from a consensus alignment of human SARS-CoV2 RNAs using RNAalifold. Figure shows nucleotides 20277-23265. Base-pair probability, representative of the breadth of the structural ensemble that could be adopted by the RNA, is shown in colour according to the key. Relates to Figure 7.
